# Mental awareness improved mild cognitive impairment and modulated gut microbiome

**DOI:** 10.18632/aging.202277

**Published:** 2020-12-09

**Authors:** Wei Wei Thwe Khine, Miao Lian Voong, Ted Kheng Siang Ng, Lei Feng, Grishma Avinash Rane, Alan Prem Kumar, Ee Heok Kua, Ratha Mahendran, Rathi Mahendran, Yuan-Kun Lee

**Affiliations:** 1 Department of Microbiology and Immunology, Yong Loo Lin School of Medicine, National University of Singapore, Singapore 117545, Singapore; 2 Functional Foods Forum, Faculty of Medicine, University of Turku, Turku 20014, Finland; 3 Department of Psychological Medicine, Yong Loo Lin School of Medicine, National University of Singapore, Singapore 117549, Singapore; 4 Department of Psychological Medicine, National University Hospital, Singapore 119228, Singapore; 5 Department of Pharmacology, Yong Loo Lin School of Medicine, National University of Singapore, Singapore 117600, Singapore; 6 Cancer Science Institute of Singapore, National University of Singapore, Singapore 117599, Singapore; 7 Medical Sciences Cluster, Yong Loo Lin School of Medicine, National University of Singapore, Singapore 117597, Singapore; 8 Department of Surgery, National University Hospital, Singapore 119228, Singapore; 9 Duke-NUS Medical School, Singapore 169857, Singapore

**Keywords:** microbiome, mental health, mild cognitive impairment, mindful awareness practice, gut-brain axis

## Abstract

There is ample scientific and clinical evidence of the effects of gut microbiota on the brain but no definitive evidence that the brain can affect changes in gut microbiota under the bi-directional gut-brain axis concept. As there is no pharmacotherapeutic intervention for the early stages of cognitive decline, research has focused on cognitive stimulation in reversing or slowing the impairment. Elderly patients diagnosed with mild cognitive impairment underwent a randomized-control trial of mindful awareness practice. Neuropsychological assessments, inflammatory markers, and gut microbiota profiles were tested. Here, we report that their cognitive impairment was improved and associated with changes in gut bacterial profile. A cognition-score-dependent-abundance was observed in *Ruminococcus* vs Recognition Trials (RT), Digit Span Backward (DSB), Semantic Fluency Span (SFS) and Memory Domain (MD); *Coprococcus* vs DSB, Color Trails Test 2 (CTT2) and Block Design (BD); *Parabacteroides* vs DSB and SFS; *Fusobacterium* vs DSB and CTT2; *Enterobacteriaceae* vs BD and SFS; *Ruminococcaceae* vs DSB; *Phascolarctobacterium* vs MD. The study showed for the first-time, alteration in the cognitive capacity leading to the corresponding changes in microbiota profiles. This strongly suggests that signals from the different segments of brain could dictate directly or indirectly the abundances of specific gut microbes.

## INTRODUCTION

With age, cognitive decline occurs along a continuum from normal aging to mild cognitive impairment (MCI) and Dementia (Major Neurocognitive Disorder, DSM 5 diagnosis) [1–3]. There is no pharmacotherapeutic cure for cognitive decline; medications that are available merely slow the process of decline but cannot reverse it. However, there is increasing evidence for the effectiveness of cognitive stimulation activities such as psychosocial interventions [4, 5], in reversing the decline.

Mindfulness is defined as “paying attention in an intentional and non-judgmental way to the present moment” [6, 7]. Although rooted in Buddhist practices, it is now modified for use in secularized interventions and is a cost-effective, acceptable, and a non-invasive approach to treat a broad spectrum of disorders [8]. We were particularly interested in mindful awareness practices for the elderly as earlier studies have demonstrated that it could enhance cognitive reserve capacity and slow down age-related cognitive decline [5, 9]. These effects appeared to be mediated by strengthened neuronal circuits [10], enhanced immune regulation [11], changes in gene expression and activity [12], reduced levels of certain inflammatory markers [13, 14] and lowered levels of oxidative stress markers leading to telomere lengthening [15].

In the complex and dynamic environment of the human gastrointestinal (GI) tract, an intricate, symbiotic relationship exists between the host and gut microbiota, with far-reaching effects at local (GI) and systemic sites (such as the brain). This underpins the concept of a bi-directional gut-brain axis [16–18] where brain signals influence gut functions and gut microbiota affect brain functioning. Evidence suggests that complex mediating factors such as neurotransmitter, inflammation and immunological dysfunction are at play at the GI level in leading to cognitive changes [1, 19–22]. On the other hand, there have been no studies to demonstrate the effects of cognitive changes, in particular, cognitive decline on the gut microbiome and whether attempts to arrest or reverse cognitive decline could change the gut microbiota profile. This would definitively establish the bi-directional nature of the gut microbiome-brain axis.

To examine the effect of the brain on gut microbiota, we first established whether there was a difference in the gut microbiota profile in Normal Aging subjects compared to the MCI patients. We then introduced a psychosocial intervention: Mindful Awareness Program (MAP), involving elderly with MCI aged 60 to 85 years, to determine if improvements in cognition were associated with changes in the gut bacteria. MAP was led weekly for 3 months and then monthly for 6 months, by an experienced instructor, involved mindfulness of the senses practice, body scan practice, and visuomotor limb tasks. MAP did not involve medication, dietary and lifestyle changes. Stool and blood samples were collected at baseline, 3 months and 9 months and represented as Timepoint 1, Timepoint 2 and Timepoint 3 respectively in this study.

In this study, we showed for the first time that alteration in the various cognitive functions among elderly MCI patients led to corresponding changes in specific microbiota abundance. In particular *Ruminococcus*, *Coprococcus*, *Parabacteroides*, *Fusobacterium*, *Enterobacteriaceae*, *Ruminocococeae* and *Phascolarcto bacterium* appeared as risk indicators of MCI.

## RESULTS

**Figure 1:**
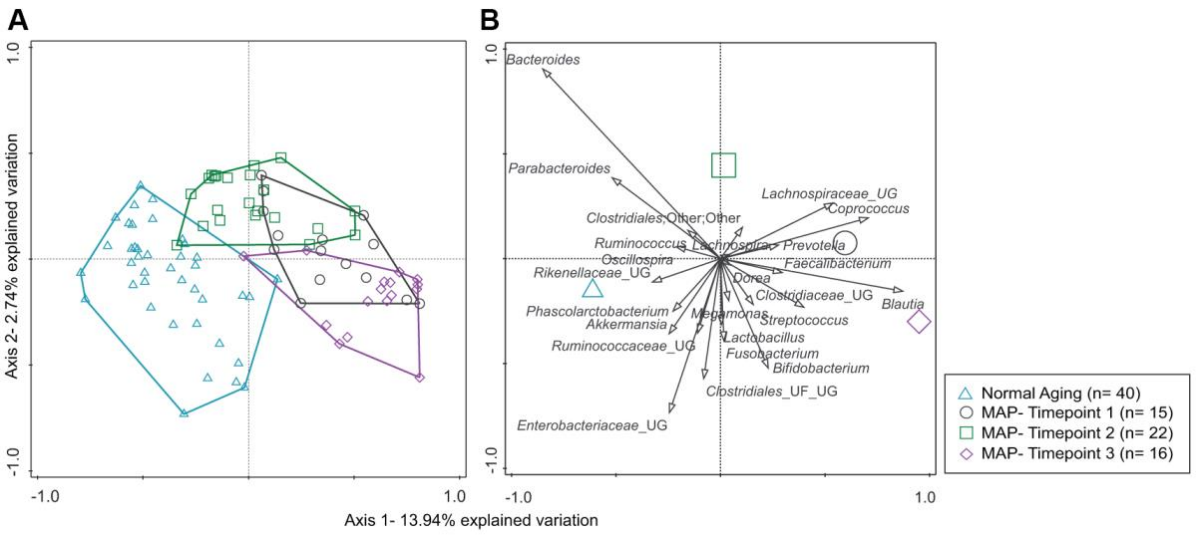
****(**A**, **B**) The distribution of microbiota profiles among Normal Aging and Mindful Awareness Program (MAP) groups. (**A**) A distance-based redundancy analysis (db-RDA) plot. (**B**) species biplot describes 1 % and above of the bacterial genera distribution in the db-RDA plane. The groups of the subjects are represented by four different color-coded symbols with sample size in parenthesis in the legend.

In this study, the fecal microbiome of the cohort of community-living elderly could be segregated into those who were aging normally from those with MCI with permutational multivariate analysis of variance (PERMANOVA) post-hoc Bonferroni multiple comparison tests (Figure 1, Supplementary Table 1) at 4999 permutations. Upon completion of 3-months of weekly MAP, distance-based redundancy analysis (db-RDA) plot of the microbiome of the MCI group (Timepoint 2) showed a visible migration away from the baseline, Timepoint 1 (Figure 1). A further 6-month monthly MAP resulted in the retraction of the plot (Timepoint 3) towards the baseline (Timepoint 1).

**Figure 2:**
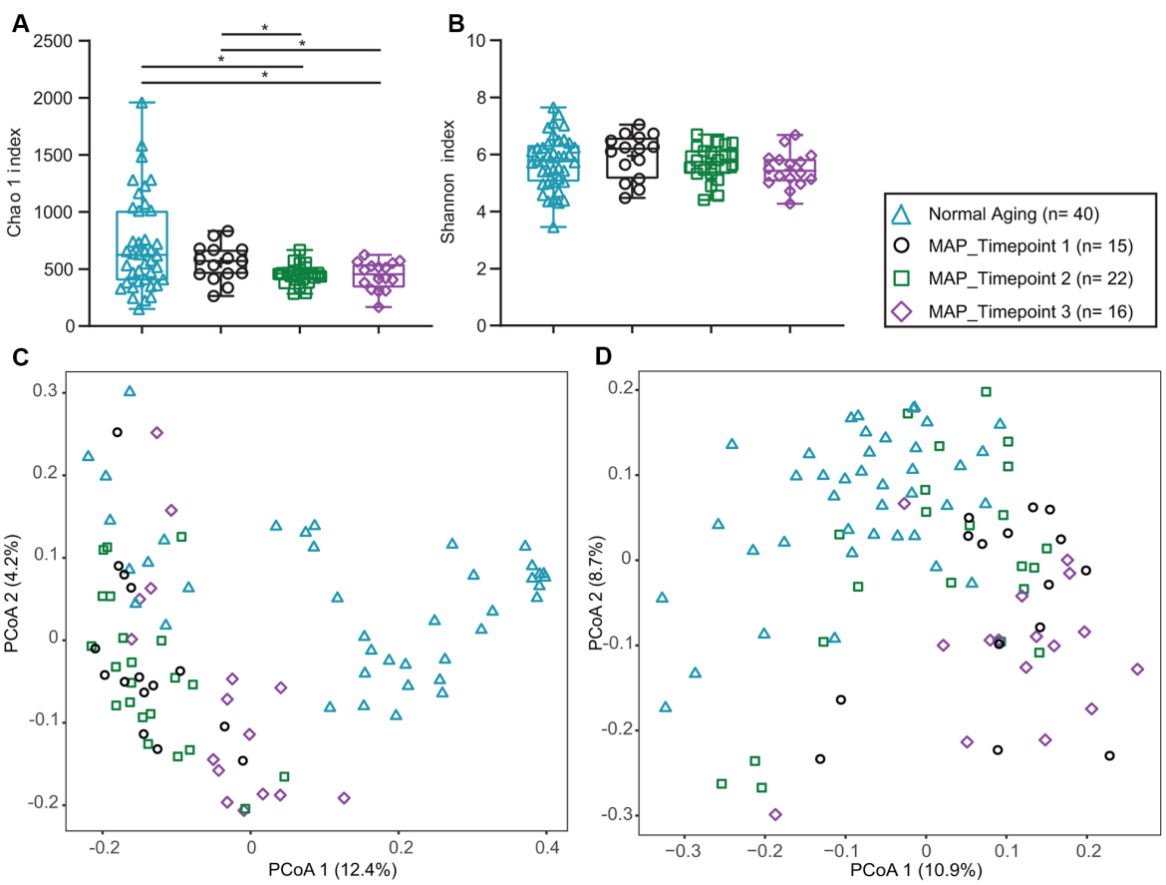
**Alpha and beta diversity of Normal Aging and MAP groups.** (**A**) Chao 1’s, (**B**) Shannon’s alpha diversity indexes comparing Normal Aging and three time points of MAP groups. p* values of Mann-Whitney U test described significant difference from each other at two-sided p values of 0.05. In each box plot, median line, + mean, upper and lower quartiles, upper and lower extremes and whiskers are presented. (**C**) Weighted (**D**) Unweighted Unifrac principal coordinates analysis (PCoA) for beta diversity comparing Normal Aging and three time points of MAP groups. The groups of the subjects are represented by four different color-coded symbols with sample size in parenthesis in the legend. MAP= Mindful Awareness Program.

The Shannon and Chao 1 alpha-diversity indexes were comparable between the normal aging and MCI groups before the MAP intervention (Figure 2A, 2B, Supplementary Table 2). These suggested that there was no difference in species richness and evenness between the two groups. On the other hand, both the unweighted (p=0.0012) and weighted (p=0.0012) Unifrac beta-diversity distances demonstrated significant differences between the Normal Aging and MAP groups (Figure 2C, 2D, Supplementary Table 3), suggesting differences in quantity and types of bacteria.

**Figure 3:**
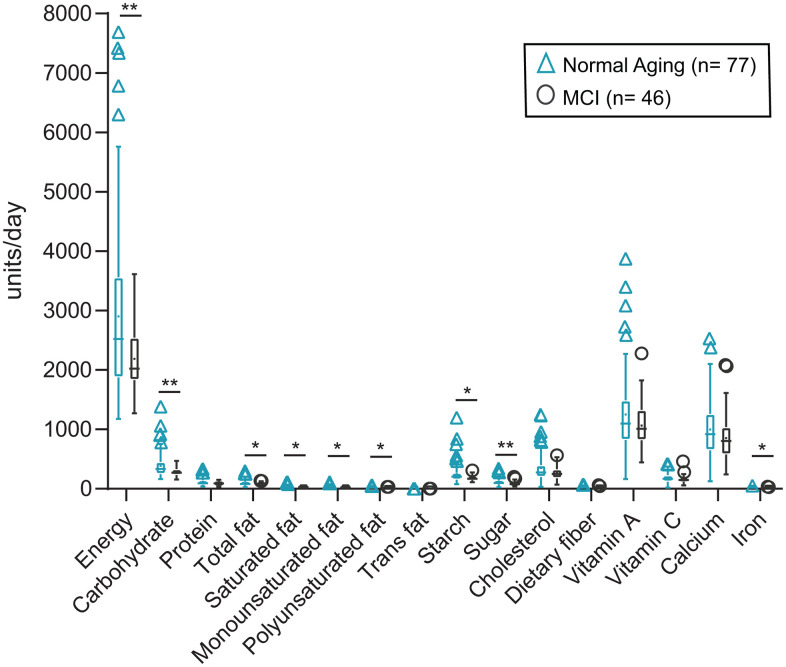
**Nutritional intake of Normal Aging and MCI subjects.** Overall nutritional intake comparing two groups of the subject. Two-tailed p values calculated by non-parametric Mann-Whitney U-test were described in the individual box plots and presented as ** p ≥ 0.001 - < 0.01, * p ≥ 0.01- < 0.05. In each box plot, median line, + mean, upper and lower quartiles, upper and lower extremes and whiskers are presented. The groups of the subjects are represented by two different color-coded symbols with sample size in parenthesis in the legend. MCI= Mild cognitive impairment.

The normal aging group of elderly consumed about 30% more frequently in all categories of macro-nutrients than the MCI patients (Figure 3, Supplementary Table 4). However, the relative proportions of the macro-, micro-nutrients consumed by each group were comparable among the different nutrients (Figure 3, Supplementary Table 5). The proportion of macro-nutrients (carbohydrates, protein, fat) relative to energy consumed by Norming Aging and MCI subjects were not distinguishable.

**Figure 4:**
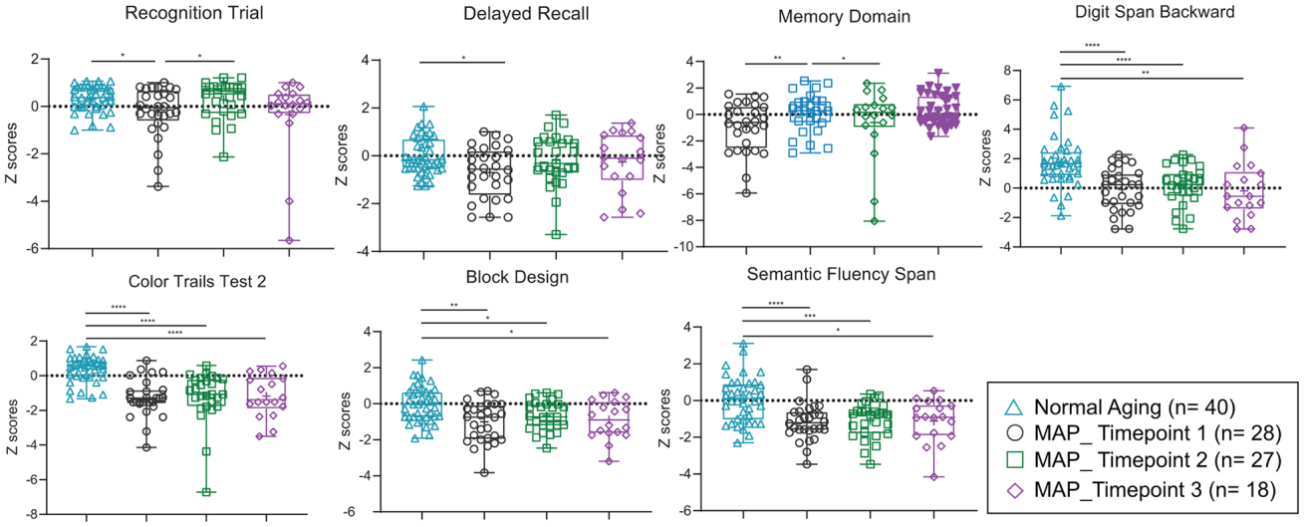
**Neuropsychological tests of MCI subjects, which showed differences with Normal Aging subjects, and during MAP intervention study.** Significant different p values (two-tailed, at p= 0.05) of Mann-Whitney U test are described comparing two groups and presented as **** p < 0.0001, *** p ≥ 0.0001 - < 0.001, ** p ≥ 0.001 - < 0.01, * p ≥ 0.01- < 0.05. In each box plot, median line, + mean, upper and lower quartiles, upper and lower extremes and whiskers are presented. The groups of the subjects represented by four different color-coded symbols with sample size are indicated in parenthesis in the legend. MCI= Mild cognitive impairment, MAP= Mindful awareness program.

All seven cognitive functions tested (Figure 4, Supplementary Table 6) were found significantly lower in MCI subjects before MAP practice. During the MAP program, improvement in some of the cognitive functions was observed after 3 months of MAP practice. Significant changes were observed in the Recognition Trials and Memory Domain but not Delayed Recall, Digit Span Backward, Color Trails Test 2, Block Design and Semantic Fluency Span cognitive functions, when guided interventions were performed weekly (Figure 4, Supplementary Table 6). Extension of the MAP intervention in the subsequent 6 months with frequency reduced to monthly guided sessions, which served as self-control in the study, resulted in the deteriorating towards that of MCI stage (MAP Timepoint 1 and 3 showed no significant differences).

**Figure 5:**
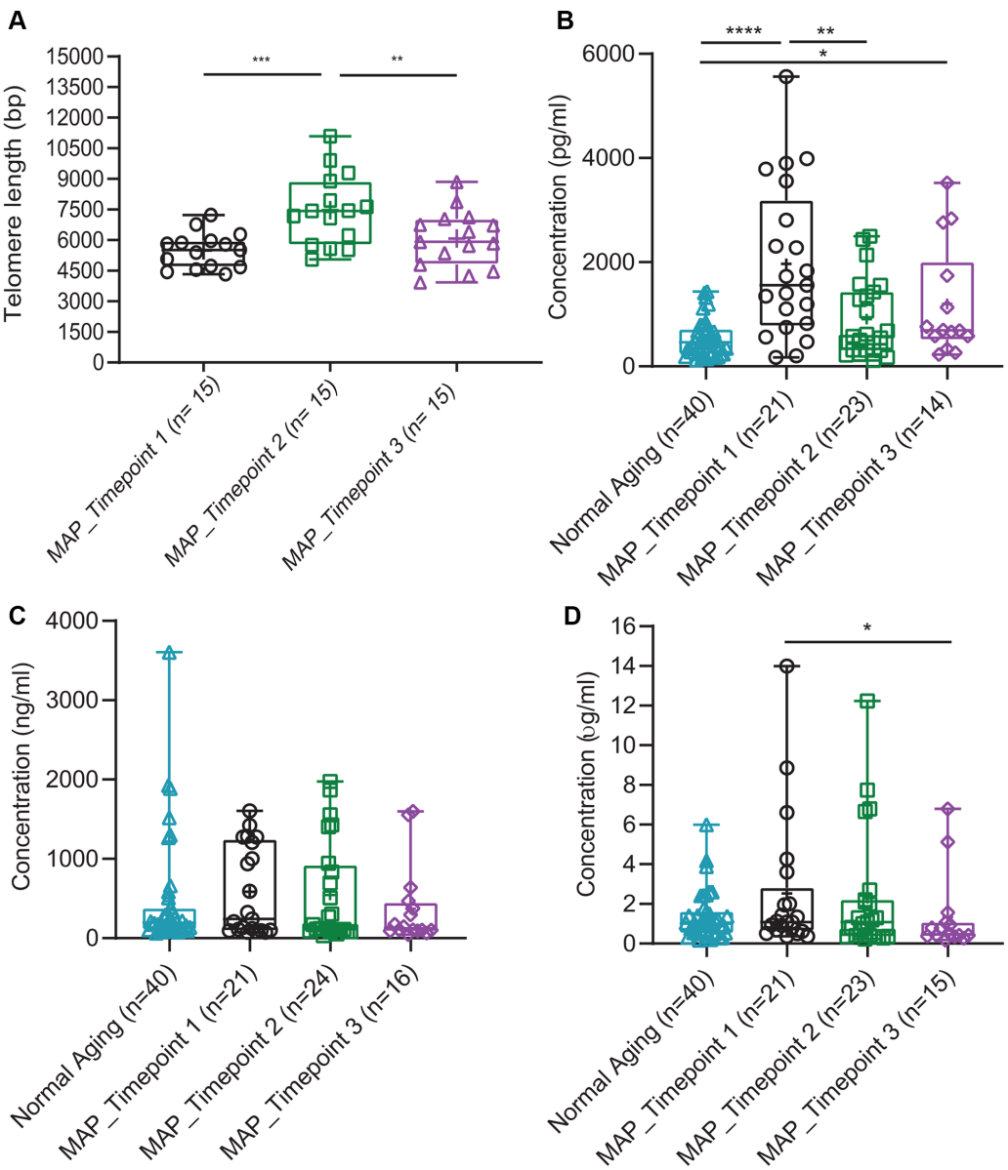
**Comparison of four blood biomarkers in Normal Aging and MAP groups.** (**A**) Mean of telomere length over time in the MAP intervention study. P values were calculated by Wilcoxon matched-pairs signed-rank t test comparing the two groups. The samples of Normal Aging were not measured for telomere length. (**B**) Concentrations of BDNF (pg/ml), (**C**) DHEAS (ng/ml) and (**D**) hs-CRP (μg/ml) comparing Normal Aging and three timepoints of MAP groups. Significant different two-tailed p values of Mann-Whitney U test are presented as **** p < 0.0001, *** p ≥ 0.0001 - < 0.001, ** p ≥ 0.001 - < 0.01, * p ≥ 0.01- < 0.05. In each box plot, median line, + mean, upper and lower quartiles, upper and lower extremes and whiskers are presented. The groups of the subjects represented by three different color-coded symbols with sample size are indicated in parenthesis in the legend. MAP= Mindful Awareness Program, BDNF= Brain-derived neurotrophic factor, DHEAS= Dehydroepiandrosterone sulfate, hs-CRP= High sensitive C-reactive protein.

The changes in cognitive functions during the intervention study were verified by the measures of telomere integrity (Figure 5A) and plasma brain-derived neurotrophic factor (BDNF) (Figure 5B). Mean telomere length (TL) was observed to significantly increase by 1659 bp (Figure 5A, p < 0.001, Supplementary Table 7) in the MAP group after three months, and significantly reduced at 9-month. Whereas, the level of plasma BDNF in the MCI group measured after three months of weekly MAP practice (Timepoint 2) approached the level of the normal aging and different from that of the normal aging group after 9 months of monthly MAP practice (Timepoint 3) (Figure 5B, Supplementary Table 8). Plasma dehydroepiand rosterone sulfate (DHEAS) (Figure 5C, Supplementary Table 8) levels measured among all the Normal Aging and MCI subjects were comparable.

**Figure 6:**
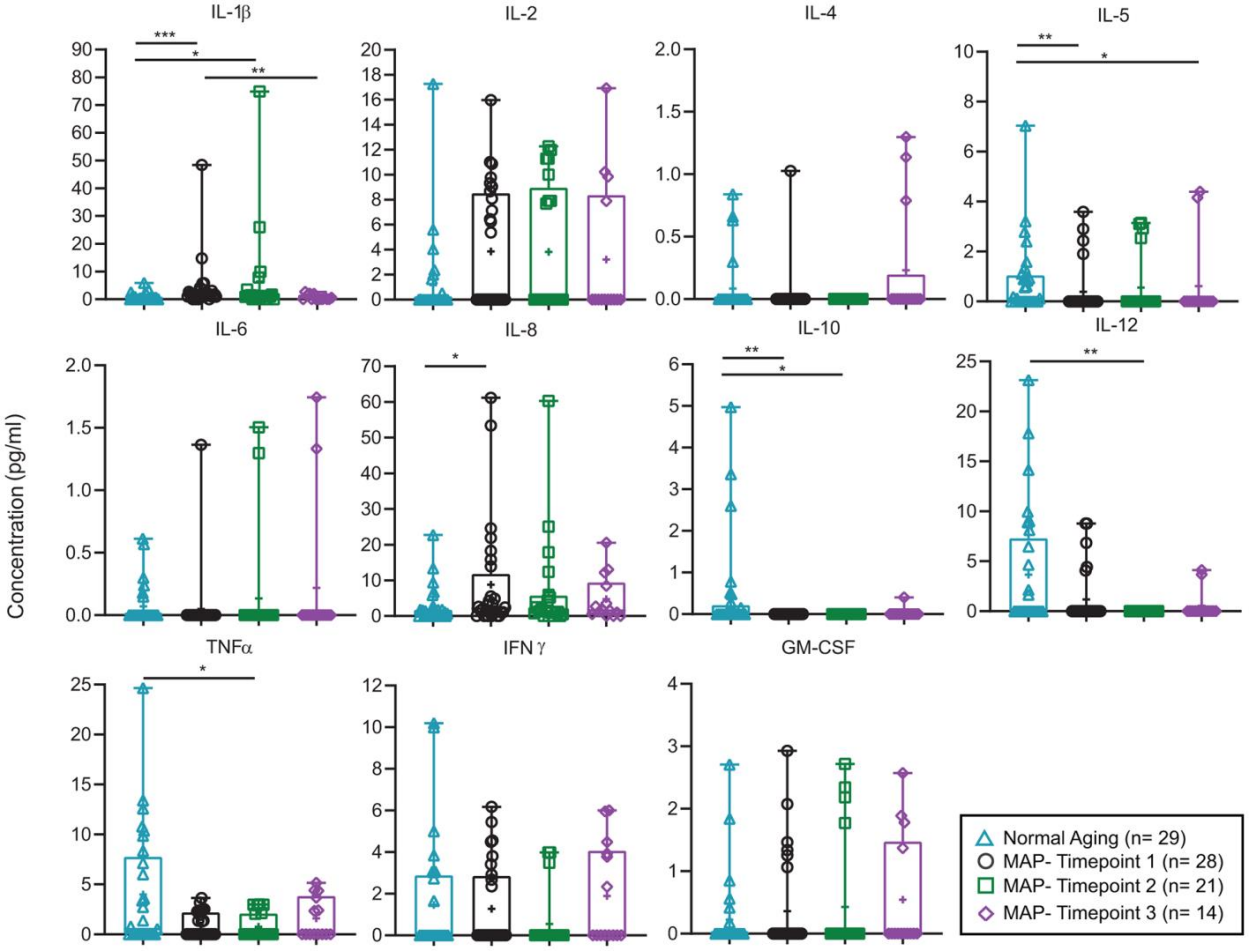
**Comparison of fecal water cytokines in Normal Aging and MAP groups.** Concentration of 11 fecal water cytokines (pg/ml) are described in each box plot comparing Normal Aging and three timepoints of MAP groups. Significant different two-tailed p values of Mann-Whitney U test are presented as **** p < 0.0001, *** p ≥ 0.0001 - < 0.001, ** p ≥ 0.001 - < 0.01, * p ≥ 0.01- < 0.05. In each box plot, median line, + mean, upper and lower quartiles, upper and lower extremes and whiskers are presented. The groups of the subjects represented by three different color-coded symbols with sample size are indicated in parenthesis in the legend. MAP= Mindful awareness program, IL= Interleukin, IL-1β= Interleukin-1 beta, TNFα= Tumor necrosis factor alpha, IFNγ= Interferon gamma, GM-CSG= Granulocyte-macrophage colony-stimulating factor.

Inflammatory markers, plasma C-Reactive Protein (CRP) (Figure 5D, Supplementary Table 8) and fecal water inflammatory cytokines IL- 1 β (Figure 6, Supplementary Table 9) were decreased significantly after 9 months of MAP (Timepoint 3) compared to the MAP Timepoint 1. However, IL- 1β of MAP at Timepoint 2 (after 3 months of intensive MAP) did not differ from Timepoint 1, suggesting improvement in cognitive functions were not related to IL- 1β level. At the same time, the rest of fecal water inflammatory cytokines such as IL- 2, -4, -5, -6, -8, -12, TNF- α, IFN- ɣ, GM- CSF and anti-inflammatory cytokine IL- 10 and plasma CRP did not alter with the cognitive stage during MAP intervention (Figure 6, Supplementary Table 9).

**Figure 7:**
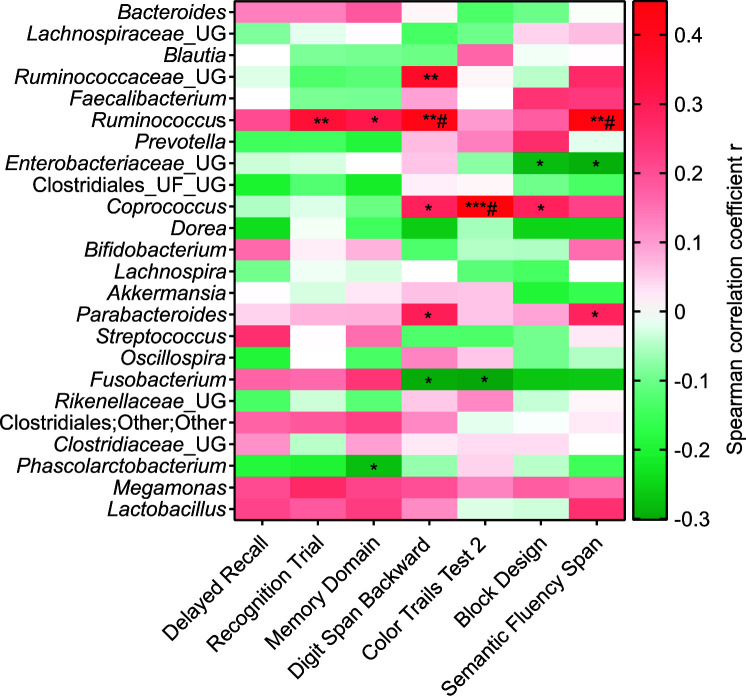
**Correlation between Z scores of neuropsychological tests and relative abundances of major gut bacterial genus (>1% of total OTU) of three timepoints of MAP groups.** In the heatmap, Spearman correlation coefficient rho (r) are presented in red (positive correlation), white (no correlation) and green (negative correlation). The significant different correlations are presented as **** p < 0.0001, *** p ≥ 0.0001 - < 0.001, ** p ≥ 0.001 - < 0.01, * p ≥ 0.01- < 0.05. The p values were corrected by false discovery rate using the Benjamini and Hochberg method and q values were represented as # q ≥ 0.01- < 0.05. OTU= Operational taxonomical unit, MAP= Mindful awareness program, UG= Unknown genus, UF= Unknown family. MAP (both correlation parameters); n= 53 (Timepoint 1; n= 28, Timepoint 2; n= 21, Timepoint 3; n= 14).

To evaluate the correlation between a cognitive function during the MAP and corresponding changes in microbiota abundance, Spearman's non-parametric correlation was adopted, for the data distribution was not in Gaussian and both variables were not controlled by the experiment. A Spearman rho (r) of ± 0.3 was considered a strong correlation. As shown in Figure 7 and Supplementary Table 10 based on two-tailed p values of Spearman test, *Ruminococcus* was positively and strongly correlated with the four cognitive functions, namely Recognition Trials, Digit Span Backward, Semantic Fluency Span and Memory Domain; whereas *Ruminocococeae* positively correlated with Digit Span Backward; *Coprococcus* positively correlated with Color Trails Test 2, Digit Span Backward and Block Design; and *Parabacteroides* positively correlated with Digit Span Backward and Semantic Fluency Span. *Enterobacteriaceae* negatively associated with Block Design and Semantic Fluency Span; *Fusobacterium* negatively correlated with Digit Span Backward and Color Trails Test 2; and *Phascolarctobacterium* negatively associated with Memory Domain. When q values were calculated using False Discovery Rate (FDR), *Ruminococcus* was found positively associated with Digit Span Backward and Semantic Fluency Span; while *Coprococcus* was positively associated with Color Trails Test 2.

## DISCUSSION

All seven cognitive functions tested showed differences between Normal Aging and MCI subjects. In the MAP intervention study, the findings indicated improvements in some of the cognitive functions, telomere integrity and plasma BDNF after 3 months of MAP practice when the guided interventions were performed weekly. The improvement in cognitive functions, telomere integrity and plasma BDNF was not sustained in some cases at 9 months when the intervention frequency was reduced to monthly after the first 3 months. This served as self-control on the frequency-dependent effects of the MAP practice. Telomere: the protective cap at the end of the chromosome, is considered a determinant of cellular aging [23, 24]. TL shortens with aging and the rate is amplified by inflammation and oxidative stress, the pre-diseases mechanisms of most age-related diseases [25–27]. The plasma BDNF is involved in numerous cognitive processes and the level of BDNF in brain is assumed to play a crucial role in the pathophysiology of mild neurocognitive disorders [28, 29]. An earlier study verified the correlation between MAP and MCI brain dysconnectivity using functional Magnetic resonance imaging (fMRI) technology [5]. All these verified direct associations between MAP practice and cognitive functions.

Interestingly, fecal water cytokines IL- 1β, -2, -4, -5, -6, -8, -12, TNF- α, IFN- ɣ, GM- CSF, inflammatory marker plasma CRP and anti-inflammatory cytokine IL-10 did not alter with the cognitive stage during MAP intervention study. These suggested that the MAP-mediated changes in cognitive functions were not mediated by inflammation in our study. Ng et al [14] however, reported that plasma IL- 1β level was reduced after 3 months of MAP practice in males.

Plasma DHEAS is the major secretory steroid of the human adrenal glands and decreases with aging [30, 31]. The comparable levels of DHEAS measured among all the Normal Aging and MCI subjects in this study suggested that changes in cognitive capacity during the MAP practice were not an aging-related process.

The distance-based redundancy analysis (db-RDA) plot of the microbiome of the Normal Aging and MCI subjects (Timepoint 1) showed clear segregation. Upon completion of 3 months of weekly MAP practice, the MCI group (Timepoint 2) showed a visible migration away from the baseline: Timepoint 1. Further 6-month monthly MAP resulted in the retraction of the plot (Timepoint 3) towards the baseline (Timepoint 1). The beta-diversity of the fecal microbiome followed a similar trend in the MAP program, suggesting that regular and frequent MAP practice led to changes in the quantity and type of microbiota.

Diet is generally accepted as the major eco-environmental factor in determining the gut microbiota profile [32–26], and the relative proportions of dietary energy, protein, fat, and carbohydrates were found to dictate the predominant gut microbiota type [32–36]. The relative proportion of the macro-nutrients (carbohydrates, protein, fat) consumed by Normal Aging and MCI subjects in this study were comparable (Supplementary Tables 4, 5). This implies that the cognitive capacity and variation in the microbiota profile were not due to the composition of their diet. This, however, did not rule out the possible effect of appetite and body weight, as the normal aging group in general, consumed foods about 30% more frequently than the MCI patients. The abundance of *Blautia, Ruminococcus* and *Akkermansia* were reported to be associated with body weight and obesity (related to food quantity consumed) [37, 38].

Further analysis of correlation between cognitive functions and respective microbiota found that only *Ruminococcus* was correlated with four cognitive functions (Recognition Trial, Memory Domain, Digit Span Backward, and Semantic Fluency Span). Six other major microbiota (>1% total OTUs), namely *Coprococcus,* *Parabacteroides, Ruminocococeae*, *Fusobacterium, Enterobacteriaceae* and *Phascolarcto bacterium*, but not *Blautia* and *Akkermansia*, which have been shown to be associated with body weight, were associated with one to three cognitive functions. It appeared that alteration in the capacity of the various cognitive functions (attributed to different parts of the brain) led to changes in the abundance of specific gut microbiota differently, thus strongly suggesting that signals from different segments (functions) of the brain could dictate directly or indirectly the abundance of certain microbes (in our case *Ruminococcus, Coprococcus,* *Parabacteroides, Ruminocococeae,* *Fusobacterium, Enterobacteriaceae* and *Phascolarcto bacterium*). At this stage, we are not able to verify the mechanism of brain-gut microbiota communication, but it is unlikely to involve inflammation and aging-related pathways.

The study opens the option of the gut microbiota as a risk indicator for MCI. Bercik [39] reported that a specific probiotic in mice might be effective and has a direct effect on their anxiety-like behavior. Gareau et al. [40] also demonstrated that probiotics given before and during an infection event could prevent memory dysfunction. Taken together, they provide tentative evidence that there is truly bidirectional communication between the gut microbiota and brain functions.

### Limitations

Human intervention studies using *Ruminococcus,* *Coprococcus,* *Parabacteroides, Ruminocococeae,* *Fusobacterium, Enterobacteriaceae* and *Phascolarcto bacterium* were not possible, for these bacteria are not in the list of approved probiotics under Singapore food regulations and laws.

## CONCLUSIONS

The concept of gut microbiota affecting the brain function, or vice-versa, is still in the infancy stage, though many direct and indirect evidence have been proposed in the last decade. Here, we showed for the first time, that elderly patients who were diagnosed with MCI had a specific microbiota distribution profile, and alteration in the cognitive impairment led to corresponding changes in microbiota profile, thus demonstrating a brain to gut microbiota direction of communication and feasibility of gut microbiome as risk indicator of MCI.

## MATERIALS AND METHODS

### Study design

A cohort of community-living elderly Chinese individuals aged 60-85 years of both genders was recruited at the Training and Research Academy (TaRA), Singapore. This avoided possible bias on the diet and lifestyle of patients in care-unit and normal aging references living at home [41]. The inclusion criteria were: elderly individuals who fulfilled the operational criteria/definition of MCI with at least, one age-education adjusted neuropsychological test Z score less than (-)1.5, did not meet Diagnostic and Statistical Manual of Mental Disorders (DSM-5) criteria for dementia [42], had memory/cognitive complaints, preferably corroborated by a reliable informant, had intact activities of daily living, did function independently and could travel on their own to the site of the MAP program. The exclusion criteria were those who had a neurological condition such as epilepsy, Parkinson’s disease, a major psychiatric condition such as major depressive disorder, significant visual or hearing impairment, color blindness, upper and lower limb motor difficulties, suffered from a terminal illness and participated in another interventional study at the same time. Some of the fecal and blood samples were excluded from specific analyses because of insufficient amount or poor quality of samples for different types of analyses. The characteristics of the participants at baseline are is presented in Table 1.

**Table 1:** Demographic characteristic of the participants at baseline.

**Characteristics**		**Normal Aging (n= 77)**	**MCI Time point 1 (n= 46)**
Mean age and range at the diagnosis (years)		65 (60-85)	67 (60-79)
Gender, No. (%)	Male	19 (24.6%)	14 (30.4%)
	Female	58 (75.3%)	32 (69.6%)
Race, No. (%)	Chinese	75 (97.4%)	43 (93.5%)
	Indian	0	2 (4.3%)
	Malay	2 (2.6%)	0
	NA	0	1 (2.2%)

MCI= Mild Cognitive Impairment.

After the neuropsychological diagnosis was made, 123 subjects (46 MCI and 77 Normal Aging subjects) participated in the study and 28 MCI patients (mean age 71.3 years, 71.4% female and 96.4% Chinese) were randomly assigned to undergo a Mindful Awareness Program (MAP) intervention for 9 months. 40 Normal Aging subjects (mean age 69 years, 72.5% female and 100% Chinese) provided stool and blood samples. The neuropsychological assessments and the stool (for gut microbiota profiling and fecal water cytokines analysis) and blood (for telomere length measurement, high sensitive C-reactive protein: hs-CRP assay, brain-derived neurotrophic factor: BDNF assay and dehydroepiandrosterone sulfate: DHEAS assay) samples collection were made at baseline (both MCI/MAP-Timepoint 1 and Normal Aging groups), at 3-months (MAP-Timepoint 2) and 9-months (MAP-Timepoint 3).

Participants in the MAP group were taught mindful awareness practice techniques: group work mindfulness-based practices for older adults [43, 44] guided by in-house trained staff. The guided interventions involved mindfulness of the senses practice (to focus/notice on the five sensory perceptions: sounds, sight, scents, taste and touch at present), body scan practice (to scan the different body parts for any sensations), walking meditation practice (to concentrate the detail process of walking movement in the cycle by walking slowly), movement nature (to observe flexibility, strength and confidence while moving the body naturally) and visuomotor limb tasks (to train the mind-body coordination) were provided by an experienced instructor and the participants continued to live as usual, without medication and change in dietary habits and lifestyle. In the initial three months, guided interventions were provided for 40 minutes weekly and the subjects practiced daily. Serving as self-control, the remaining six months of guided practices were conducted for 45 minutes monthly instead.

This study was approved by the Institutional Review Board of the National University of Singapore, Singapore. Informed consent from all subjects was obtained before participation and all experiments were performed according to the standard operating procedures and guidelines.

### Food frequency questionnaire data requisition and analysis

A food frequency questionnaire (FFQ) (Supplementary Text 1) from the 2004 report of the national nutrition survey, health promotion board, Singapore [45] was given to each participant to record their food intake 24 hours before their fecal sample collection. A total of 123 subjects (77 Normal Aging and 46 MCI) reported FFQs. The FFQ captures the frequency of consumption per day/week/month and portion size of food items such as bread/cereals, rice/porridge, noodles, soups, vegetables, bean curd, dressings, fruits, poultry, meat, fish, eggs, desserts, biscuits/pastries, fast foods, beverages, nuts/snacks, milk/dairy products, soy products, and alcoholic drinks. The information captured were converted to the total frequency of food item consumed per day and then, to the total portion size of foods consumed per day. It was computed to energy and nutrient composition of foods by an online tool of energy and nutrient composition of food, from the Health Promotion Board^©^, Singapore [46] so that it reflected the energy and nutrient composition amount consumed per day.

### Cognitive assessment

A modified version of the original Mini-Mental State Examination (MMSE, range 0-30) [47] was administered to all the subjects as a measure of cognitive function. Participants who obtained a MMSE score lower than pre-specified cut-off values (≤ 27 for subjects without formal education, ≤ 28 for primary school education level, ≤ 29 for secondary school and above.) were invited to the study center for further neurocognitive assessment. To determine the signs of cognitive impairment, clinical history, Clinical Dementia Rating (CDR) [48–50], a battery of standard neuropsychological tests (Supplementary Text 2) (Rey Auditory Verbal Learning Test; RAVLT [51–53], Digit Span [54], Block Design [54, 55], Color Trails Test [56, 57] and Semantic Fluency Span [58–60]), Geriatric Depression Scale (GDS) [61] and Geriatric Anxiety Index (GAI) [62, 63] were assessed. These observations and scores were reviewed by a panel of clinical consultants before a formal diagnosis of the patients’ mental state could be made by consensus opinion. The neuropsychological diagnosis was made using the Petersen's criteria of MCI and local norms (age and education adjusted) of the neuropsychological tests [64]. The assessments were conducted on all the participants throughout all three timepoints. In this report, quantitative evaluation of RAVLT (Delayed Recall, Recognition Trial, Memory Domain), Digit Span Backward, Block Design, Color Trails Test 2 and Semantic Fluency Span are included. In this study, 40 Normal Aging subjects and 28 (MAP-Timepoint 1), 27 (MAP-Timepoint 2), 18 (MAP-Timepoint 3) MCI patients completed the neuropsychological tests.

### Blood samples collection

Five ml of blood were collected in the ethylenediaminetetraacetic acid (EDTA) tube between 09:00 and 11:00 after overnight fasting and not in strenuous physical activities [65, 66] from 40 Normal Aging subjects and 28 (MAP-Timepoint 1), 27 (MAP-Timepoint 2), 18 (MAP-Timepoint 3) MCI patients for telomere length measurement, hs-CRP, BDNF and DHEAS assays. Blood samples were kept at 4° C for three hours at most temporarily. Plasma samples were extracted by centrifugation of 4 ml of whole blood at 1650 x g for 25 minutes at room temperature. Plasma and 1 ml of non-centrifuged blood were stored at -80° C until further analysis.

### Telomere length measurement

Blood samples from 15 MCI subjects who had undergone three-time points of MAP were used to analyse telomere length measurement. Genomic DNA was extracted from 100 ul anticoagulated blood samples using DNeasy blood and tissue kit (Qiagen, Co, Hilden, Germany). Telomere Restriction Fragment (TRF) length analysis followed by southern blot was used to estimate average telomere length (TL) (TeloTTAGG telomere length assay kit, Roche Diagnostics, Mannheim, Germany) following the manufacturer’s protocol with some modifications [67]. Briefly, 1 μg of DNA was digested using Hinf I/Rsa I enzymes at 37° C for 2 hours. The digested product was resolved on 0.8% agarose gel. The gel was washed in depurination solution (0.25M HCL) for 30 mins followed by two washes with denaturation solution (0.5 M NaOH, 1.5 M NaCl), 20 mins each, and then incubated in a neutralization solution (0.5 M Tris–HCl, 3 M NaCl, pH 7.5) for 20 mins, twice. The digested DNA was then transferred onto a nylon membrane (Hybond- N^+^, Amersham, UK) overnight by capillary osmosis in 20X SSC (3 M NaCl, 0.3 M sodium citrate tribasic dehydrate, pH 7). The DNA was fixed by UV-cross-linking at 120 mJ using a Stratalinker^®^ UV Crosslinker (Stratagene) and hybridization was performed at 42° C with digoxigenin-labeled telomeric probe. The TRF smear was detected using a digoxigenin luminescent detection system and the smear signal was recorded on X-ray films which were then digitized. Average TL was estimated by comparison to a 1kb plus DNA ladder using TeloTool software (Matlab, The MathWorks, Inc., Natick, MA, USA) [68].

### Plasma hs-C-reactive Protein (CRP), Brain-derived Neurotrophic factor (BDNF) and Dehydroepian drosterone Sulfate (DHEAS) assays

hs-CRP, BDNF and DHEAS assays were measured from 10, 10 and 100 μl of plasma using enzyme-linked immunosorbent assay (ELISA) kits of Tecan trading (Männedorf, Switzerland), Promega Co. (Madison, USA) and CUSABIO tech. (Houston, USA) respectively. All the experiments were performed by following the respective manufacturer’s protocol and the absorbance was measured at 450 nm using the microplate reader. In this study, the results of 40 Normal Aging subjects and 21 (MAP-Timepoint 1), 24 (MAP-Timepoint 2), 16 (MAP-Timepoint 3) MCI patients could be reported and the exact sample size could be found in Figure 5.

### Gut microbiota profiling analysis

### Fecal samples processing and DNA extraction

Approximately 1 g of fecal samples preserved in 2 ml of RNAlater^®^ (Ambion, Inc., Texas, USA) were collected from 40 Normal Aging subjects and 28 (Timepoint 1), 27 (Timepoint 2), 18 (Timepoint 3) MCI subjects for gut microbiota profiling analysis. Among three time points of MAP, 15, 22 and 18 samples for Timepoint 1, 2 and 3 respectively, were able to proceed for 16s rRNA gene sequencing after quantification and qualification check. 0.2 ml of fecal homogenate was washed with Phosphate-buffered saline (PBS) for two times. DNA was extracted using the phenol-chloroform method. After treated with the Tris-SDS solution, the mixture was mixed with TE-saturated phenol (Sigma-Aldrich, Cor., St. Louis, Missouri, USA) and then, the glass-beads mechanically extraction method was applied. The supernatant was added into phenol/chloroform/isoamyl alcohol (25:24:1) which was homogenized again. Sodium acetate and isopropanol precipitated the DNA followed by washing with 70% ethanol. Once the pellet was dry, DNA was eluted in TE buffer.

### 16s rRNA gene sequencing

The concentration of double-stranded DNA was quantified using Quanti-iT™ PicoGreen^®^ dsDNA kit (Invitrogen, Inc., Carlsbad, USA). Before Polymerase Chain Reaction (PCR), DNA was normalized to a concentration of 12.5 ng. The normalized DNA was amplified using KAPA HiFi™ HotStart ReadyMix kit (Roche life science, Inc., Indiana, USA) and a primer set that was targeted for amplification at v3 and v4 regions of the 16s rRNA. Following amplicon PCR, the amplicons were purified using Agencourt^®^ AMPure XP beads (Beckman Coulter, Inc., Fullerton, CA, USA) and suspended in 10 mM Tris buffer. For the addition of indices and adapter sequences to the amplicons, the second round of PCR was set up using Nextera XT index primers and KAPA HiFi HotStart ReadyMix. After that, the DNA was purified again with Agencourt AMPure XP beads and eluted in 10 mM Tris buffer. To ensure the quality and the size of base pairs of the library, Agilent High Sensitivity DNA kit (Agilent Technologies, Inc., CA, USA) was used in Agilent 2100 Bioanalyzer (Agilent Technologies, Inc., CA, USA).

The library was quantified with Quanti-iT™ PicoGreen^®^ dsDNA kit (Invitrogen, Inc., Carlsbad, USA). The individual library was then, normalized to a concentration of 4 nM with 10 mM Tris buffer. The normalized libraries were pooled to become a pooled amplicon library (PAL). PAL was quantified using the KAPA library quantification kit (Roche life science, Inc., Indiana, USA) in the Applied Biosystems™ ABI 7500 Real-Time PCR system (Thermo Fisher Scientific, Inc., Massachusetts, USA). PAL was denatured with 0.2 N NaOH and mixed with hybridization buffer (HT1) (Illumina, Inc., San Diego, USA) to further diluted until the final loading concentration of 6-8 pM. The diluted denatured library was spiked with the denatured diluted PhiX control library and sequenced in the Miseq system (Illumina, Inc., San Diego, USA).

### Bioinformatics analysis

The 16s rRNA DNA sequence data were analyzed using Quantitative Insights Into Microbial Ecology (QIIME) version 1.9.1 [69]. Corresponding reverse and forward reads were first joined, and the resultant paired reads were quality filtered at least a Q-score of 25. Chimeric sequences were also filtered out and removed using USEARCH v6.1 [70]. The resultant sequences were then picked out to the operational taxonomic unit (OTU), using 97% similarity sequences in Greengenes v13_8 database and open reference OTU picking method. The OTUs were then summarized into taxa to further elucidate the bacterial profile of the samples. Here, we used bacterial genera for further data analysis. A constrained ordination of distance-based redundancy analysis (db-RDA) was performed using the relative abundances of genus-level data and the Bray Curtis distance in the Canoco5 software (Microcomputer Power Co, Ithaca, USA) and species biplot was constructed.

### Fecal water cytokines analysis

In this analysis, the fecal water samples from 29 Normal Aging subjects and 28 (MAP-Timepoint 1), 21 (MAP-Timepoint 2), 14 (MAP-Timepoint 3) MCI patients were involved. 0.01 M Phenylmethylsulfonyl fluoride (PMSF) (Roche Diagnostics, Mannheim, Germany) and 1% Bovine Serum Albumin (BSA) treated 200 μl of fecal homogenate was assayed for the inflammatory and anti-inflammatory cytokines: Interleukin (IL)-1β, - 2, -4, -5, -6, -8, -12, TNF- α, IFN- ɣ, GM- CSF and IL- 10 using LUNARIS^TM^ Human 11-Plex cytokine kit (AYOXXA Biosystems, Austria). The experiments were performed according to the manufacturer’s protocol and the fluorescence was measured by Zeiss Axio Imager M2 (Carl Zeiss, Oberkochen, Germany) and quantified with the LUNARIS^TM^ analysis.

### Data and statistical analysis

All the graphs and statistical analyses were done by GraphPad Prism 8.1 software (GraphPad, Inc., San Diego, USA), Canoco 5 (Microcomputer Power, USA) and R 3.5.2 software (RStudio, Inc., Boston, USA). Among bacterial genera, only the top 1% of relative abundance were used. Normality tests were performed on 1% of genus data, alpha diversity index, neuropsychological scores, nutrients data, and four blood biomarkers data (telomere lengths, BDNF, DHEAS, hs-CRP and fecal water cytokines). For db-RDA analysis (Figure 1), Monte Carlo permutation test was calculated comparing the dis-similarities in the distribution of microbiota using the Bray-Curtis distance matrix tested on all possible axes for all data points and resulted in pseudoF= 6.2 and p= 0.0002. For beta diversity analysis (Figure 2C, 2D), principal coordinates analysis (PCoA) plots were drawn comparing the dis-similarities in the distribution of microbiota for the weighted and unweighted unifrac distances using phyloseq [71] and ggplot2 [72] R packages. The statistical test of permutational multivariate analysis of variance (PERMANOVA) with 4999 permutations followed by post-hoc Bonferroni multiple comparison test pairwiseAdonis R package [73]. The package calculated the sums of squared, F model, R^2^, p and adjusted p values (Supplementary Tables 1 and 3). The unpaired, non-parametric Man-Whitney U test was performed pairwise comparison for alpha diversity, nutrients, neuropsychological Z scores, BDNF, DHEAS, hs-CRP and fecal water cytokines between normal aging and MCI groups and between different time points of MAP. For the comparison of telomere data between the groups, the non-parametric Wilcoxon matched-pairs signed-rank test was performed. All the p values are calculated at two-tailed and confidence interval at 95%. Moreover, to examine the correlations between neuropsychological Z scores and the abundances of the major bacterial genus (>1% of total bacteria), non-parametric Spearman rho and two-tailed p values were calculated. The p values were corrected by false discovery rate using the Benjamini and Hochberg method, resulting in the q values.

### Availability of data and materials

The dataset generated and analyzed for this study are available in the paper, the supplementary information in the supplementary files, protocol exchange (2019) DOI: 10.21203/rs.3.pex-342/v1 and EBI repository (accession no: PRJEB32675). Materials and data should be addressed to the corresponding authors: Rathi Mahendran (pcmrathi@nus.edu.sg) and Yuan-Kun Lee (micleeyk@nus.edu.sg).

### Ethics approval

This study was approved by the Institutional Review Board of the National University of Singapore, Singapore (NUS-IRB Ref: No. 10-517, NUS-IRB Ref: No. 13-168 and NUS-IRB Ref: No. B14-110). The study record has been registered at ClinicalTrials.gov ID no of NCT02286791. Informed consent was obtained by all individuals before participation and all experiments were performed by approved guidelines and regulations.

## Supplementary Material

Supplementary Tables

Supplementary Text 1

Supplementary Text 2
