# Supplementary material for: Mental awareness improved mild cognitive impairment and modulated gut microbiome
Source: Aging (Albany NY). 2020 Dec 9;12(23):24371–93. doi: 10.18632/aging.202277 (PMC7762482; doi:10.18632/aging.202277)
Supplement: Supplementary Tables [file aging-12-202277-s001.pdf]

SUPPLEMENTARY TABLES

Supplementary Table 1. Results of the permutational multivariate analysis of variance (PERMANOVA) test comparing MCI and Normal Aging groups.

Pairs	Sums of Squared	F.Model	R^2	Method- Adonis, Bonferroni test	
				p.value	p.adjusted
Normal Aging vs MAP_TP1	1.087745	8.338268	0.135939	0.0002***	0.0012**
Normal Aging vs MAP_TP2	0.675926	5.531065	0.084404	0.0002***	0.0012**
Normal Aging vs MAP_TP3	1.811868	12.74869	0.190995	0.0002***	0.0012**
MAP_TP1 vs MAP_TP2	0.318482	3.307593	0.086343	0.0046**	0.0276*
MAP_TP1 vs MAP_TP3	0.188403	1.471758	0.048299	0.1166	0.6996
MAP_TP2 vs MAP_TP3	0.828449	7.22011	0.167054	0.0002***	0.0012**

Sums of squared, F model, R^2, p and adjusted p values shown were calculated from the pairwise comparison by the statistical test of PERMANOVA and 4999 permutations based on the Bray-Curtis distance of the relative abundances of bacteria. p and adjusted p values were corrected by Bonferroni test and presented as *** p ≥ 0.0001 - < 0.001, ** p ≥ 0.001 - < 0.01, * p ≥ 0.01- < 0.05. MAP= Mindful Awareness Program, TP1= Timepoint 1, TP2= Timepoint 2, TP3= Timepoint 3. Normal Aging; n= 40, MAP_TP1; n= 15, MAP_TP2; n= 22, MAP_TP3; n= 16.

Supplementary Table 2. Results (p values) of the statistical test of Chao 1 and Shannon of alpha diversity comparing the MAP and Normal Aging groups in pair.

Pairs	Two-tailed p values	
	Chao 1	Shannon
Normal Aging vs MAP_TP1	0.4826	0.3156
Normal Aging vs MAP_TP2	0.0113*	0.9709
Normal Aging vs MAP_TP3	0.0177*	0.2448
MAP_TP1 vs MAP_TP2	0.0356*	0.2019
MAP_TP1 vs MAP_TP3	0.0405*	0.0784
MAP_TP2 vs MAP_TP3	0.9651	0.2342

Two-tailed p values were calculated from the statistical test of unpaired, non-parametric, Mann Whitney U test based on Chao 1 and Shannon indices of alpha-diversity. * indicates that one variable is significantly different from other counterpart at p < 0.05. MAP= Mindful Awareness Program, TP1= Timepoint 1, TP2= Timepoint 2, TP3= Timepoint 3. Normal Aging; n= 40, MAP_TP1; n= 15, MAP_TP2; n= 22, MAP_TP3; n= 16.

Supplementary Table 3. Results of permutational multivariate analysis of variance (PERMANOVA) of the gut microbiota using Unifrac distance.

Pairs	Sums of Squared		F.Model		R^2		p.value		p.adjusted	
	Weighted Unifrac	Unweighted Unifrac	Weighted Unifrac	Unweighted Unifrac	Weighted Unifrac	Unweighted Unifrac	Weighted Unifrac	Unweighted Unifrac	Weighted Unifrac	Unweighted Unifrac
Normal Aging vs MAP_TP1	0.66	1.23	4.05	4.28	0.07	0.07	0.0002***	0.0002***	0.0012**	0.0012**
Normal Aging vs MAP_TP2	0.53	1.42	3.32	5.14	0.05	0.08	0.0002***	0.0002***	0.0012**	0.0012**
Normal Aging vs MAP_TP3	0.86	0.99	5.17	3.48	0.09	0.06	0.0002***	0.0002***	0.0012**	0.0012**
MAP_TP1 vs MAP_TP2	0.21	0.36	1.53	1.42	0.04	0.04	0.0576	0.002**	0.3456	0.012*
MAP_TP1 vs MAP_TP3	0.17	0.46	1.16	1.77	0.04	0.06	0.2114	0.0006***	1	0.0036
MAP_TP2 vs MAP_TP3	0.44	0.53	3.02	2.11	0.08	0.06	0.0002***	0.0002***	0.0012**	0.0012**

Sums of squared, F model, R^2, p and adjusted p values shown were calculated from the pairwise comparison by the statistical test of PERMANOVA and 4999 permutations based on weighted and unweighted Unifrac distances of the abundances of bacteria. p values and adjusted p values were corrected by Bonferroni test. The significantly differences of pairs are presented as *** p ≥ 0.0001 - < 0.001, ** p ≥ 0.001 - < 0.01, * p ≥ 0.01- < 0.05. MAP= Mindful Awareness Program, TP1= Timepoint 1, TP2= Timepoint 2, TP3= Timepoint 3. Normal Aging; n= 40, MAP_TP1; n= 15, MAP_TP2; n= 22, MAP_TP3; n= 16.

Supplementary Table 4. Ratio of average nutrients consumption between Normal Aging and MCI.

Nutrients	Ratio of average nutrient consumption (Normal Aging/ MCI)
Energy	1.327409
Carbohydrate	1.337354
Protein	1.323174
Total fat	1.30519
Saturated fat	1.287847
Monounsaturated fat	1.311511
Polyunsaturated fat	1.334471
Trans fat	1.220319
Starch	1.395529
Sugar	1.264992
Cholesterol	1.348599
Dietary fiber	1.195017
Vitamin A	1.176913
Vitamin C	1.177098
Calcium	1.168511
Iron	1.278677

Ratio of average nutrients consumption between normal aging and MCI were calculated by average nutrients consumption of normal aging group over that of MCI group. The data applied, here, were before Mindful Awareness Program (MAP). MCI= Mild Cognitive Impairment. Normal Aging; n= 77, MCI; n= 46.

Supplementary Table 5. Fraction of average nutrients consumption relative to energy.

Nutrients/Energy	Normal Aging	MCI
Energy/Energy	1	1
Carbohydrate/Energy	0.13	0.13
Protein/Energy	0.04	0.04
Total fat/Energy	0.03	0.03
Saturated fat/Energy	0.01	0.01
Monounsaturated fat/Energy	0.01	0.01
Polyunsaturated fat/Energy	0.01	0.01
Trans fat/Energy	1.03	<0.01
Starch/Energy	0.09	0.08
Sugar/Energy	0.04	0.04
Cholesterol/Energy	0.12	0.12
Dietary fiber/Energy	0.01	0.01
Vitamin A/Energy	0.43	0.49
Vitamin C/Energy	0.06	0.07
Calcium/Energy	0.34	0.39
Iron/Energy	0.01	0.01

Fractions of average nutrients consumption were calculated using the same denominator of energy of each group. MCI= Mild Cognitive Impairment. Normal Aging; n= 77, MCI; n= 46.

Supplementary Table 6. Results (p values) of the statistical test of neuropsychological tests comparing the MCI and Normal Aging groups in pair.

Neuropsychological tests	Recognition Trial	Delayed Recall	Memory Domain	Digit Span Backward	Color Trails Test 2	Block Design	Semantic Fluency Span
Normal Aging vs MAP_TP1	0.022*	0.0176*	0.008**	<0.0001****	<0.0001****	0.001**	<0.0001****
Normal Aging vs MAP_TP2	0.729	0.557	0.847	<0.0001****	<0.0001****	0.0108*	0.0002***
Normal Aging vs MAP_TP3	0.148	0.871	0.761	0.0002***	<0.0001****	0.0084*	0.0024*
MAP_TP1 vs MAP_TP2	0.0306*	0.104	0.0365*	0.480	0.272	0.260	0.528
MAP_TP1 vs MAP_TP3	0.529	0.137	0.291	0.536	0.836	0.612	0.620
MAP_TP2 vs MAP_TP3	0.153	0.904	0.642	0.271	0.770	0.504	0.995

Two-tailed p values were calculated from the statistical test of unpaired, non-parametric, Mann Whitney U test for the neuropsychological tests. The significantly differences of pairs are presented as **** $p < 0.0001$, *** $p \geq 0.0001$ - < 0.001, ** $p \geq 0.001$ - < 0.01, * $p \geq 0.01$- < 0.05. MAP= Mindful Awareness Program, TP1= Timepoint 1, TP2= Timepoint 2, TP3= Timepoint 3. Normal Aging; n= 40, MAP_TP1; n= 28, MAP_TP2; n= 27, MAP_TP3; n= 18.

Supplementary Table 7. Results (p values) of the statistical test of telomere length measured at different intervention points of MAP comparing the timepoints in pair.

Pairs	p values
Timepoint 1 vs. Timepoint 2	0.0001***
Timepoint 1 vs. Timepoint 3	0.3303
Timepoint 2 vs. Timepoint 3	0.0067**

Two-tailed p values were calculated from the statistical test of paired, non-parametric, Wilcoxon matched-pair signed rank test based on the telomere length of MAP comparing the different time points. The significantly differences of pairs are presented as *** $p \geq 0.0001$ - < 0.001, ** $p \geq 0.001$ - < 0.01. MAP= Mindful Awareness Program. n= 15 for each timepoints.

Supplementary Table 8. Results (p values) of the statistical test of three plasma biomarkers comparing the MAP and Normal Aging groups in pair.

Pairs	(1) BDNF	(2) DHEAS	(3) hs-CRP
Normal Aging vs MAP_TP1	<0.0001****	0.7348	0.3065
Normal Aging vs MAP_TP2	0.093	0.7673	0.7154
Normal Aging vs MAP_TP3	0.0169*	0.3373	0.1486
MAP_TP1 vs MAP_TP2	0.0076**	0.6768	0.4738
MAP_TP1 vs MAP_TP3	0.0959	0.3385	0.0351*
MAP_TP2 vs MAP_TP3	0.2567	0.5032	0.2999

Two-tailed p values were calculated from the statistical test of unpaired, non-parametric, Mann Whitney U test for three plasma biomarkers. The significantly differences of pairs are presented as **** p < 0.0001, *** p ≥ 0.0001 - < 0.001, ** p ≥ 0.001 - < 0.01, * p ≥ 0.01- < 0.05. BDNF= Brain derived neurotrophic factor, DHEAS= Dehydroepiandrosterone sulfate, hs-CRP= high sensitive C-reactive protein, MAP= Mindful Awareness Program, TP1= Timepoint 1, TP2= Timepoint 2, TP3= Timepoint 3. Normal Aging (n= 40,40,40), MAP_TP1 (n= 21,21,21), MAP_TP2 (n= 23,24,23), MAP_TP3 (n= 14,16,15).

Supplementary Table 9. Results (p values) of the statistical test of fecal water cytokines comparing the MAP and Normal Aging groups in pair.

Pairs	IL-1β	IL-2	IL-4	IL-5	IL-6	IL-8	IL-10	IL-12	TNFα	IFNγ	GM-CSF
Normal Aging vs MAP_TP1	0.0001***	0.0704	0.3525	0.0065**	0.102	0.0342*	0.0045**	0.0664	0.0862	0.9405	0.7697
Normal Aging vs MAP_TP2	0.0181*	0.1878	0.1291	0.0536	0.441	0.09	0.0147*	0.0016**	0.0404*	0.2418	0.9315
Normal Aging vs MAP_TP3	0.6581	0.683	0.4117	0.0457*	0.915	0.203	0.1289	0.0764	0.3665	0.3822	0.4695
MAP_TP1 vs MAP_TP2	0.2437	0.9508	>0.9999	0.5148	0.463	0.845	>0.9999	0.0622	0.6327	0.1669	>0.9999
MAP_TP1 vs MAP_TP3	0.0066**	0.5938	0.0545	0.6922	0.465	0.624	0.3333	0.6685	0.3813	0.468	0.5118
MAP_TP2 vs MAP_TP3	0.0971	0.5897	0.0556	>0.9999	0.757	0.907	0.4	0.1529	0.2265	0.0617	0.6473

Two-tailed p values were calculated from the statistical test of unpaired, non-parametric, Mann Whitney U test for 11 fecal water cytokines. The significantly differences of pairs are presented as *** p ≥ 0.0001 - < 0.001, ** p ≥ 0.001 - < 0.01, * p ≥ 0.01- < 0.05. MAP= Mindful Awareness Program, TP1= Timepoint 1, TP2= Timepoint 2, TP3= Timepoint 3, IL= Interleukin, IL-1β= Interleukin-1 beta, TNFα= Tumor necrosis factor alpha, IFNγ= Interferon gamma, GM-CSG= Granulocyte-macrophage colony-stimulating factor. Normal Aging; n= 29, MAP_TP1; n= 28, MAP_TP2; n= 21, MAP_TP3; n= 14.

Supplementary Table 10. Results of correlation between fecal bacterial genus abundance (>1% total OTUs) and Z scores of neuropsychological tests among all MAP groups.

Spearman r correlation coefficient	Delayed Recall	Recognition Trial	Memory Domain	Digit Span Backward	Color Trails Test 2	Block Design	Semantic Fluency Span
Bacteroides	0.13	0.13	0.18	0.01	-0.13	-0.10	-0.01
*Lachnospiraceae*_UG	-0.08	-0.02	0.01	-0.14	-0.10	0.04	0.06
Blautia	0.00	-0.09	-0.09	-0.10	0.16	-0.01	0.01
*Ruminococcaceae*_UG	-0.02	-0.13	-0.12	0.37**	0.01	-0.05	0.27
Faecalibacterium	0.00	-0.09	-0.09	0.09	0.00	0.25	0.24
Ruminococcus	0.21	0.35**	0.31*	0.42**#	0.10	0.18	0.44**#
Prevotella	-0.15	-0.14	-0.19	0.07	0.13	0.26	-0.02
*Enterobacteriaceae*_UG	-0.04	-0.03	0.00	0.06	-0.08	-0.28*	-0.29*
Clostridiales_UF_UG	-0.21	-0.12	-0.22	0.02	0.01	-0.10	-0.14
Coprococcus	-0.05	-0.02	-0.11	0.29*	0.45***#	0.29*	0.22
Dorea	-0.24	-0.01	-0.15	-0.26	-0.06	-0.25	-0.25
Bifidobacterium	0.16	0.02	0.07	-0.13	-0.05	-0.05	0.15
Lachnospira	-0.10	-0.01	-0.03	0.00	-0.12	-0.14	0.00
Akkermansia	0.01	-0.03	0.03	0.06	0.06	-0.19	-0.16
Parabacteroides	0.05	0.08	0.08	0.29*	0.06	0.09	0.28*
Streptococcus	0.26	0.00	0.15	-0.13	-0.13	-0.10	0.02
Oscillospira	-0.19	0.00	-0.14	0.13	0.06	-0.09	-0.05
Fusobacterium	0.17	0.16	0.24	-0.30*	-0.30*	-0.27	-0.26
*Rikenellaceae*_UG	-0.14	-0.04	-0.12	0.05	0.12	-0.04	0.01
Clostridiales;Other;Other	0.17	0.19	0.23	0.12	-0.02	-0.01	0.02
*Clostridiaceae*_UG	0.11	-0.05	0.09	0.03	0.04	0.04	0.00
Phascolarctobacterium	-0.19	-0.20	-0.28*	-0.07	0.05	-0.05	-0.15
Megamonas	0.21	0.27	0.22	0.20	0.13	0.18	0.15
Lactobacillus	0.22	0.18	0.23	0.12	-0.03	-0.03	0.25

Spearman rho (r) values derived from non-parametric Spearman correlation test were described. Three timepoints of MAP were included in calculation of correlation between 1% of fecal bacterial genus and Z scores of neuropsychological tests. The significantly different correlations are represented as *** p ≥ 0.0001 - < 0.001, ** p ≥ 0.001 - < 0.01, * p ≥ 0.01- < 0.05. The two-tailed p values were corrected by false discovery rate using the Benjamini and Hochberg method and q values are represented as # q ≥ 0.01- < 0.05. MAP= Mindful Awareness Program, UG= Unknown genus, UF= Unknown family.
