# Supplementary material for: Mental awareness improved mild cognitive impairment and modulated gut microbiome
Source: Aging (Albany NY). 2020 Dec 9;12(23):24371–93. doi: 10.18632/aging.202277 (PMC7762482; doi:10.18632/aging.202277)

Supplementary Text 1: Food Frequency Questionnaires

# For Research Staffs

Subject Number: ___________________

**Interviewer**: _______________________ Date of Interview: _________________

Signature: _________________________

**Reviewer**: _________________________ Date of Review: ___________________

Signature: _________________________

Remarks (if any):

**Data-entry staff** ____________________ Date of Data-entry: ___________________

Signature: ________________________

Remarks (if any):

The questionnaires are adopted from Singapore National Nutrition Survey 2010 with permission from the Health Promotion Board (HPB), Singapore.

**Part A**

1. Have you changed your diet in the past one month? Yes / No*

1. If yes, why did you do so?

_______________________________________________________________________________________________________________________________________________________________________________________________________________

1. What were the changes you made?

_______________________________________________________________________________________________________________________________________________________________________________________________________________

*Please delete whichever is not applicable

**PART B**

1. What type of oil/fat do you/your family use for cooking (pan frying, deep frying, stewing)? Choose from the list.
   1. Blended vegetable oil (cooking oil)
   2. Polyunsaturated oil (corn, soya, sunflower, safflower, grapeseed, flaxseed)
   3. Monounsaturated oil (olive, peanut, canola, rice bran, sesame, mustard)
   4. Saturated fat (lard, ghee, tallow, hard margarine, butter, shortening, coconut oil, palm oil)
   5. Do not pan fry, deep fry or stew.
2. What type of oil/fat do you/your family use for cooking (stir frying)? Choose from list.
   1. Blended vegetable oil (cooking oil)
   2. Polyunsaturated oil (corn, soya, sunflower, safflower, grapeseed, flaxseed)
   3. Monounsaturated oil (olive, peanut, canola, rice bran, sesame, mustard)
   4. Saturated fat (lard, ghee, tallow, hard margarine, butter, shortening, coconut oil, palm oil)
   5. Do not stir fry
3. What type of oil/fat do you/your family use for baking/roasting? Choose from list.
   1. Blended vegetable oil (cooking oil)
   2. Polyunsaturated oil (corn, soya, sunflower, safflower, grapeseed, flaxseed)
   3. Monounsaturated oil (olive, peanut, canola, rice bran, sesame, mustard)
   4. Saturated fat (lard, ghee, tallow, hard margarine, butter, shortening, coconut oil, palm oil)
   5. Do not bake or roast
4. What type of milk do you use with your coffee? Choose from the list.
5. Creamer
6. Sweetened condensed milk
7. Evaporated milk
8. Full cream milk/powder
9. Low fat milk/powder
10. Skimmed milk/powder
11. No added milk
12. Whitener
13. Do not drink coffee

E. What type of milk do you use with your tea? Choose from the list.

1. Creamer
2. Sweetened condensed milk
3. Evaporated milk
4. Full cream milk/powder
5. Low fat milk/powder
6. Skimmed milk/powder
7. No added milk
8. Whitener
9. Do not drink tea

F. What type of milk do you use with malt beverages? Choose from the list.

1. Creamer
2. Sweetened condensed milk
3. Evaporated milk
4. Full cream milk/powder
5. Low fat milk/powder
6. Skimmed milk/powder
7. No added milk
8. Whitener
9. Do not drink malt beverages

G. Do you usually ask for less sugar/“less sweet” when ordering beverages?

1. No
2. Yes

H. Do you usually ask for less sugar/“less sweet” when ordering desserts?

0. No

1. Yes

**Breads and Cereals**

| **Food Item** | **Portion** | **Number of times eaten** | | | |
| --- | --- | --- | --- | --- | --- |
| **How often do you eat the following:** |  | **Per day** | **Per week** | **Per month** | **Rarely/Never** |
| **Bread** | |  |  |  |  |
| 1. White bread, including naan | 1 slice or 1 piece |  |  |  |  |
| 1. Wholemeal bread | 1 slice or 1 piece |  |  |  |  |
| 1. Bread with fruits and nuts | 1 slice or 1 piece |  |  |  |  |
| **Bread spreads used** | |  |  |  |  |
| 1. Butter | 1 tsp (D2) |  |  |  |  |
| 1. Margarine | 1 tsp (D2) |  |  |  |  |
| 1. Peanut butter | 1 tsp (D2) |  |  |  |  |
| 1. Jams/honey | 1 tsp (D2) |  |  |  |  |
| 1. Kaya | 1 tsp (D2) |  |  |  |  |
| **Other types of breads** |  |  |  |  |  |
| 1. Roti prata/murtabak | 1 piece |  |  |  |  |
| 1. Chapati/thosai | 1 piece |  |  |  |  |
| 1. French toast/roti telur/roti john | 1 piece |  |  |  |  |
| 1. Bread buns with coconut/curry/meat fillings | 1 piece |  |  |  |  |
| **Cereals** |  |  |  |  |  |
| 13. Plain/flavoured breakfast cereals | 4 dsp (D1) |  |  |  |  |
| 14. Mixed (with fruit/nuts) breakfast cereals | 4 dsp (D1) |  |  |  |  |
| ***For participants who consume breakfast cereals (#13, 14):*** | | | | | |
| *4001. You have indicated that you eat breakfast cereals. How often do you have breakfast cereals made from whole-grains?* | 4 dsp (D1) |  |  |  |  |
| 3001. Oats/oatmeal (raw) | 4 dsp (D1) |  |  |  |  |

**Rice and Porridge**

| **Food Item** | **Portion** | **Number of times eaten** | | | |
| --- | --- | --- | --- | --- | --- |
| **How often do you eat the following:** |  | **Per day** | **Per week** | **Per month** | **Rarely/Never** |
| 15. Plain rice | 1 B1 |  |  |  |  |
| ***For participants who consume plain rice (#15):*** | | | | | |
| *4002. You have indicated that you eat plain rice. How often do you have rice prepared using brown or red rice?* | 1 B1 |  |  |  |  |
| 16. Plain porridge | 1 B1 |  |  |  |  |
| ***For participants who consume plain porridge (#16):*** | | | | | |
| *4003. You have indicated that you eat plain porridge. How often do you have porridge prepared using brown or red rice?* | 1 B1 |  |  |  |  |
| **Flavoured rice/porridge** | | | | | |
| 17. Fried rice | 1 B1 |  |  |  |  |
| 18. Chicken/duck rice | 1 portion |  |  |  |  |
| 19. Mui fan | 1 portion |  |  |  |  |
| 20. Nasi briyani | 1 portion |  |  |  |  |
| 21. Nasi lemak | 1 portion |  |  |  |  |
| 22. Claypot rice | 1 portion |  |  |  |  |
| 23. Glutinuous rice | 1 portion |  |  |  |  |
| 24. Flavoured porridge (e.g. chicken, pork, duck, fish) | 1 portion |  |  |  |  |

**Noodles (rice noodles, wheat noodles, bean noodles, pasta)**

| **Food Item** | **Portion** | **Number of times eaten** | | | |
| --- | --- | --- | --- | --- | --- |
| **How often do you eat the following:** |  | **Per day** | **Per week** | **Per month** | **Rarely/Never** |
| **Soup noodles** |  |  |  |  |  |
| 25. Fishball/yong tau foo/wanton/ prawn/beef/chicken/ fish slice | 1 portion |  |  |  |  |
| ***For participants who consume soup noodles (#25):*** | | | | | |
| *4004. You have indicated that you eat noodles in soup. How often do you have soup noodles prepared using brown rice beehoon?* | 1 portion |  |  |  |  |
| 26. Penang laksa | 1 portion |  |  |  |  |
| **Dry noodles** |  |  |  |  |  |
| 27. Fishball/yong tau foo/wanton/ minced meat & mushrooms/prawn/ beef/chicken | 1 portion |  |  |  |  |
| **Fried noodles** |  |  |  |  |  |
| 29. Fried kway teow with cockles | 1 portion |  |  |  |  |
| 30. Fried hor fun | 1 portion |  |  |  |  |
| 31. Fried noodles (incl. Hokkien mee, mee goreng) | 1 portion |  |  |  |  |
| 32. Fried beehoon | 1 portion |  |  |  |  |
| ***For participants who consume fried beehoon (#32):*** | | | | | |
| *4005. You have indicated that you eat fried beehoon. How often do you have fried beehoon prepared using brown rice beehoon?* | 1 portion |  |  |  |  |
| **Noodles in gravy** |  |  |  |  |  |
| 28. Lor mee/mee rebus | 1 portion |  |  |  |  |
| 33. Laksa lemak | 1 portion |  |  |  |  |
| 34. Mee siam (with coconut milk) | 1 portion |  |  |  |  |
| **Other noodles** |  |  |  |  |  |
| 35. Instant noodles | 1 portion |  |  |  |  |
| 905. Boiled noodles / spaghetti / pasta (plain) | 1 portion |  |  |  |  |
| 906. Boiled noodles / spaghetti / pasta with tomato sauce | 1 portion |  |  |  |  |
| 907. Boiled noodles / spaghetti / pasta with cream white sauce | 1 portion |  |  |  |  |
| ***For participants who consume boiled spaghetti/pasta:*** | | | | | |
| *4006. You have indicated that you eat boiled spaghetti/pasta. How often do you have spaghetti/pasta prepared using wholemeal spaghetti/pasta?* | 1 portion |  |  |  |  |

**Soups**

| **Food Item** | **Portion** | **Number of times eaten** | | | |
| --- | --- | --- | --- | --- | --- |
| **How often do you eat the following:** |  | **Per day** | **Per week** | **Per month** | **Rarely/Never** |
| 600. Cream soup | 1 B2 |  |  |  |  |
| 601. Clear soup/broth | 1 B2 |  |  |  |  |

# Vegetables and Beancurd

| **Food Item** | **Venue** | **Portion** | **Number of times eaten** | | | |
| --- | --- | --- | --- | --- | --- | --- |
| **How often do you eat the following:** |  |  | **Per day** | **Per week** | **Per month** | **Rarely/Never** |
| **Pale green leafy vegetables (cabbage, pak choy, lettuce, beansprouts, cauliflower etc)** | | | | | | |
| 1. Stir fried , plain |  | ½ cup |  |  |  |  |
| 1. Stir fried, with meat/ seafood |  | ½ cup |  |  |  |  |
| 1. Stir fried in oyster sauce |  | ½ cup |  |  |  |  |
| 1. Curry/lemak |  | ½ cup |  |  |  |  |
| 1. Raw/steamed/in soup | ----- | 1 cup |  |  |  |  |
| **Dark green leafy vegetables (spinach, kai lan, chye sim, kangkong broccoli etc)** | | | | | | |
| 1. Stir fried , plain |  | ½ cup |  |  |  |  |
| 1. Stir fried, with meat/ seafood |  | ½ cup |  |  |  |  |
| 1. Stir fried in oyster sauce |  | ½ cup |  |  |  |  |
| 1. Stir fried in sambal belacan/dried prawns |  | ½ cup |  |  |  |  |
| 1. Raw/steamed/in soup | ----- | 1 cup |  |  |  |  |
| **Tomatoes, carrots, red/yellow peppers** | | |  |  |  |  |
| 1. Stir fried, plain |  | ½ cup |  |  |  |  |
| 1. Stir fried, with meat/ seafood |  | ½ cup |  |  |  |  |
| 1. Curry/lemak |  | ½ cup |  |  |  |  |
| 1. Raw/steamed/in soup | ----- | 1 cup |  |  |  |  |
| **Legumes/pulses, e.g. beans, peas** | | |  |  |  |  |
| 1. Stir fried, plain |  | ½ cup |  |  |  |  |
| 1. Stir fried in oyster sauce |  | ½ cup |  |  |  |  |
| 1. Stir fried in sambal belacan |  | ½ cup |  |  |  |  |
| 1. Dried legumes (e.g.dhal, dried beans) in gravy |  | ½ cup |  |  |  |  |
| 354. Raw/steamed/boiled | ----- | ½ cup |  |  |  |  |
| **Mixed vegetables** | | |  |  |  |  |
| 1. Stir fried, plain |  | ½ cup |  |  |  |  |
| 1. Stir fried, with meat/ seafood |  | ½ cup |  |  |  |  |
| 1. Stir fried in oyster sauce |  | ½ cup |  |  |  |  |
|  |  |  | **Per day** | **Per week** | **Per month** | **Rarely/Never** |
| 700. Vegetables battered deep- fried (e.g. tempura) |  | 1 serving |  |  |  |  |
| 1. Curry/lemak |  | ½ cup |  |  |  |  |
| 115. Raw/steamed/in soup/ Chinese rojak | ----- | 1 cup or 1 serving |  |  |  |  |
| **Tofu/beancurd** | | |  |  |  |  |
| 116. Fried |  | ½ square |  |  |  |  |
| 1. Steamed/ in soups | ----- | ½ square |  |  |  |  |
| **Roots/stems (potatoes, sweet potatoes, corn etc)** | | |  |  |  |  |
| 349. Stir fried potatoes |  | 1 cup |  |  |  |  |
| 1. Curry lemak |  | 1 cup |  |  |  |  |
| 1. Soups with meat stock | ----- | 1 cup |  |  |  |  |
| 1. Stews |  | 1 cup |  |  |  |  |
|  |  |  |  |  |  |  |
| 704. Preserved vegetables (chye sim, olives etc) | ----- | 1 D1 |  |  |  |  |

Salad dressings

| **Food Item** | **Portion** | **Number of times eaten** | | | |
| --- | --- | --- | --- | --- | --- |
| **How often do you have the following:** |  | **Per day** | **Per week** | **Per month** | **Rarely/Never** |
| 1. Creamy dressing –   regular (thousand island, mayonnaise, salad cream etc) | 2 dsp (D1) |  |  |  |  |
| 1. Creamy dressing – light/low fat | 2 dsp (D1) |  |  |  |  |
| 1. Oil-based dressing | 2 dsp (D1) |  |  |  |  |

**Fruits**

| **Food Item** | **Portion** | **Number of times eaten** | | | |
| --- | --- | --- | --- | --- | --- |
| **How often do you eat the following:** |  | **Per day** | **Per week** | **Per month** | **Rarely/Never** |
| 1. Orange/red/yellow fresh fruits | 1 serving* |  |  |  |  |
| 1. Other fresh fruits | 1 serving* |  |  |  |  |
| 3002. Fresh fruit juice | 1 cup |  |  |  |  |
| 1. Bananas | 1 medium* |  |  |  |  |
| 1. Durians | 5 seeds |  |  |  |  |
| 1. Canned fruits | ½ cup (M1) |  |  |  |  |
| 800. Mixed fruits (dried) | 1 serving* |  |  |  |  |

**Poultry**

| **Food Item** | **Venue** | **Portion** | **Number of times eaten** | | | |
| --- | --- | --- | --- | --- | --- | --- |
| **How often do you eat the following:** |  |  | **Per day** | **Per week** | **Per month** | **Rarely/Never** |
| **Poultry - without skin** |  |  |  |  |  |  |
| 1. Stir fried |  | 1 serving |  |  |  |  |
| 1. Pan/deep fried |  | 1 serving |  |  |  |  |
| 1. Coconut curry |  | 1 serving |  |  |  |  |
| 1. Curry without coconut |  | 1 serving |  |  |  |  |
| 1. Stew/braised/roasted |  | 1 serving |  |  |  |  |
| 1. Steamed | ----- | 1 serving |  |  |  |  |
| **Poultry - with skin** |  |  |  |  |  |  |
| 1. Stir fried |  | 1 serving |  |  |  |  |
| 1. Pan/deep fried |  | 1 serving |  |  |  |  |
| 1. Coconut curry |  | 1 serving |  |  |  |  |
| 1. Curry without coconut |  | 1 serving |  |  |  |  |
| 1. Stew/braised/roasted |  | 1 serving |  |  |  |  |
| 1. Steamed | ----- | 1 serving |  |  |  |  |

**Meat**

| **Food Item** | **Venue** | **Portion** | **Number of times eaten** | | | |
| --- | --- | --- | --- | --- | --- | --- |
| **How often do you eat the following:** |  |  | **Per day** | **Per week** | **Per month** | **Rarely/Never** |
| **Meat - lean** |  |  |  |  |  |  |
| 1. Stir fried |  | 1 serving |  |  |  |  |
| 1. Pan/deep fried |  | 1 serving |  |  |  |  |
| 1. Coconut curry/rendang |  | 1 serving |  |  |  |  |
| 1. Curry without coconut |  | 1 serving |  |  |  |  |
| 1. Stewed/braised |  | 1 serving |  |  |  |  |
| 1. Roast/grilled/BBQ |  | 1 serving |  |  |  |  |
| 1. Steamed/soup | ----- | 1 serving |  |  |  |  |
| **Meat - lean and fat** |  |  |  |  |  |  |
| 1. Stir fried |  | 1 serving |  |  |  |  |
| 1. Pan/deep fried |  | 1 serving |  |  |  |  |
| 1. Coconut curry/rendang |  | 1 serving |  |  |  |  |
| 1. Curry without coconut |  | 1 serving |  |  |  |  |
| 1. Stewed/braised |  | 1 serving |  |  |  |  |
| 1. Roast/grilled/BBQ |  | 1 serving |  |  |  |  |
| 1. Steamed/soup | ----- | 1 serving |  |  |  |  |
|  |  |  | **Per day** | **Per week** | **Per month** | **Rarely/Never** |
| **Meat – preserved/cured** |  |  |  |  |  |  |
| Sausages |  | One |  |  |  |  |
| Ham |  | 1 slice |  |  |  |  |
| Bacon |  | 1 slice |  |  |  |  |
| Canned (e.g. luncheon meat, corned beef) |  | Size of 4 square of chocolate |  |  |  |  |
| Liver and other innards |  | Size of 4 square of chocolate |  |  |  |  |

**Fish/Seafood**

| **Food Item** | **Venue** | **Portion** | **Number of times eaten** | | | |
| --- | --- | --- | --- | --- | --- | --- |
| **How often do you eat the following:** |  |  | **Per day** | **Per week** | **Per month** | **Rarely/Never** |
| **Fish** |  |  |  |  |  |  |
| 3003. Raw (e.g. sashimi) | ----- | 1 serving |  |  |  |  |
| 1. Stir fried/pan fried/deep fried |  | 1 serving |  |  |  |  |
| 1. Deep fried with batter |  | 1 serving |  |  |  |  |
| 1. Steamed |  | 1 serving |  |  |  |  |
| 1. Assam pedas |  | 1 serving |  |  |  |  |
| 1. Coconut curry |  | 1 serving |  |  |  |  |
| 255. Curry without coconut |  | 1 serving |  |  |  |  |
| 1. Grilled |  | 1 serving |  |  |  |  |
| 3004. Canned (e.g. tuna) | ------ | 1 dsp (D1) |  |  |  |  |
| **Other seafood** |  |  |  |  |  |  |
| 1. Stir fried/pan fried/deep fried |  | 1 serving |  |  |  |  |
| 1. Deep fried with batter |  | 1 serving |  |  |  |  |
| 1. Steamed |  | 1 serving |  |  |  |  |
| 1. Assam pedas |  | 1 serving |  |  |  |  |
| 1. Coconut curry |  | 1 serving |  |  |  |  |
| 1. Curry without coconut |  | 1 serving |  |  |  |  |
| 1. Grilled |  | 1 serving |  |  |  |  |

**Eggs**

| **Food Item** | **Venue** | **Portion** | **Number of times eaten** | | | |
| --- | --- | --- | --- | --- | --- | --- |
| **How often do you eat the following:** |  |  | **Per day** | **Per week** | **Per month** | **Rarely/Never** |
| **Whole eggs (including salted and century eggs)** |  |  |  |  |  |  |
| 1. Boiled/poached/in soup/steamed | ------ | 1 egg |  |  |  |  |
| 1. Fried/scrambled |  | 1 egg |  |  |  |  |
| **Egg whites, only** |  |  |  |  |  |  |
| 751. Boiled/poached/in soup/steamed | ------ | 1 |  |  |  |  |
| 752. Fried/scrambled |  | 1 |  |  |  |  |

# Desserts/Local Snacks

| **Food Item** | **Portion** | **Number of times eaten** | | | |
| --- | --- | --- | --- | --- | --- |
| **How often do you eat the following:** |  | **Per day** | **Per week** | **Per month** | **Rarely/Never** |
| **Desserts in soup** |  |  |  |  |  |
| 1. With coconut milk/cream (e.g. pulot hitam, bubor cha cha) | 1B1 |  |  |  |  |
| 1. Without coconut milk (e.g cheng tng, green bean soup, tau suan) | 1B1 |  |  |  |  |
| **Kueh kueh - steamed** |  |  |  |  |  |
| 1. With coconut/coconut milk /coconut cream (e.g kueh sarlat, kueh dadar, putu mayam, idli) | 1 piece |  |  |  |  |
| 1. Without coconut milk (kueh tutu, soon kway) | 1 piece |  |  |  |  |
| **Others** |  |  |  |  |  |
| 1. Fried snacks (e.g. you tiao, goreng pisang, Indian rojak) | 1 piece |  |  |  |  |
| 1. Dim sum – steamed (e.g chee cheong fun, dumplings, rice dumplings) | 1 serving |  |  |  |  |
| 1. Dim sum – fried/deep fried (e.g. fried carrot cake, wanton, char siew puff) | 1 piece |  |  |  |  |
| 1. Sweet Indian snacks  (e.g. burfi, halwa) | 1 piece |  |  |  |  |

# Biscuits, Pastries and Cakes

| **Food Item** | **Portion** | **Number of times eaten** | | | |
| --- | --- | --- | --- | --- | --- |
| **How often do you eat the following:** |  | **Per day** | **Per week** | **Per month** | **Rarely/Never** |
| 1. Plain biscuits | 2 pieces |  |  |  |  |
| 1. Cream filled biscuits/shortbread | 2 pieces |  |  |  |  |
| 1. Puff/flaky pastries (croissants, baked curry puffs etc) | 1 piece |  |  |  |  |
| 1. Plain butter cake/fruit cake | 1 piece |  |  |  |  |
| 1. Sponge cakes | 1 piece |  |  |  |  |
| 1. Cream cakes | 1 piece |  |  |  |  |

# Fast Foods

| **Food Item** | **Portion** | **Number of times eaten** | | | |
| --- | --- | --- | --- | --- | --- |
| **How often do you eat the following:** |  | **Per day** | **Per week** | **Per month** | **Rarely/Never** |
| 1. Burgers, with beef or chicken | 1 serving |  |  |  |  |
| 1. Burgers, fish | 1 serving |  |  |  |  |
| 1. French fries | 1 small serving |  |  |  |  |
| 1. Pizza | 2 slices |  |  |  |  |
| 1100. Mashed potato with gravy | 1 regular |  |  |  |  |

**Sweetened beverages**

| **Food Item** | **Portion** | **Number of times eaten** | | | |
| --- | --- | --- | --- | --- | --- |
| **How often do you have the following:** |  | **Per day** | **Per week** | **Per month** | **Rarely/Never** |
| 1. Sweetened beverages (e.g. soft drinks, packet drinks, yoghurt drinks) | 1 G2 |  |  |  |  |

# Nuts

| **Food Item** | **Portion** | **Number of times eaten** | | | |
| --- | --- | --- | --- | --- | --- |
| **How often do you eat the following:** |  | **Per day** | **Per week** | **Per month** | **Rarely/Never** |
| **All types of nuts** |  |  |  |  |  |
| 1. Dry roasted | ½ M1 or 1 small pkt |  |  |  |  |
| 1. Fried | ½ M1 or 1 small pkt |  |  |  |  |

# Titbits/Snacks

| **Food Item** | **Portion** | **Number of times eaten** | | | |
| --- | --- | --- | --- | --- | --- |
| **How often do you eat the following:** |  | **Per day** | **Per week** | **Per month** | **Rarely/Never** |
| 1. Fried salty snacks (crisps, prawn crackers, keropok, salted biscuits etc) | 1 small packet or equivalent |  |  |  |  |
| 1. Ice cream | 1 scoop |  |  |  |  |
| 1. Chocolate | 4 squares |  |  |  |  |

# Milk Beverages

| Food Item | **Portion** | **Number of times eaten** | | | |
| --- | --- | --- | --- | --- | --- |
| **How often do you have the the following:** |  | **Per day** | **Per week** | **Per month** | **Rarely/Never** |
| 1300. Coffee* | 1 M1 –D/2 tsp |  |  |  |  |
| 1. Tea* | 1 M1 –D/2 tsp |  |  |  |  |
| 1. Malt beverages (e.g. hot chocolate, Horlicks®, Milo®, Ovaltine®) | 1 M1 –D/2 tsp |  |  |  |  |

| **Food Item** | **Portion** | **Number of times eaten** | | | |
| --- | --- | --- | --- | --- | --- |
| **How often do you have the following:** |  | **Per day** | **Per week** | **Per month** | **Rarely/Never** |
| 353. Sugar | 1 tsp (D2) |  |  |  |  |

*Assume no sugar added

Milk & Dairy Products

| **Food Item** | **Portion** | **Number of times eaten** | | | |
| --- | --- | --- | --- | --- | --- |
| **How often do you have the following:** |  | **Per day** | **Per week** | **Per month** | **Rarely/Never** |
| **Milk ( as a drink)** |  |  |  |  |  |
| 1. Full cream milk* (fresh, UHT, powder) | 1 G2* |  |  |  |  |
| 1. Low fat milk* (fresh, UHT, powder) | 1 G2* |  |  |  |  |
| 1. Skimmed milk* (fresh, UHT, powder) | 1 G2* |  |  |  |  |
| **Yoghurt** |  |  |  |  |  |
| 1. Regular | 1 G1 |  |  |  |  |
| 1. Low fat (including frozen yoghurt) | 1 G1 |  |  |  |  |
| **Cheese** |  |  |  |  |  |
| 1. Cheese/cheese spread | 1 slice/4dsp |  |  |  |  |
| 1. Low fat cheese | 1 slice |  |  |  |  |

*this could be liquid milk or powdered milk made up to the same amount using instructions on tin.

Soya Products

| **Food Item** | **Portion** | **Number of times eaten** | | | |
| --- | --- | --- | --- | --- | --- |
| **How often do you have the following:** |  | **Per day** | **Per week** | **Per month** | **Rarely/Never** |
| 1200. Soya milk (fresh/packet/can) | 1 G2 |  |  |  |  |
| 1201. Soya beancurd (tau huay) | 1 B1 |  |  |  |  |

Vegetarian (Chinese)

| **Food Item** | **Portion** | **Number of times eaten** | | | |
| --- | --- | --- | --- | --- | --- |
| **How often do you have the following:** |  | **Per day** | **Per week** | **Per month** | **Rarely/Never** |
| 1. . Fried vegetarian kway   teow/beehoon/mee/rice | 1 portion |  |  |  |  |
| 1. Gluten (char siew/duck) | 1 piece |  |  |  |  |
| 1. . Fried beancurd sheet | 1 piece |  |  |  |  |

**Alcoholic drinks**

| **Food Item** | **Portion** | **Number of times eaten** | | | |
| --- | --- | --- | --- | --- | --- |
| **How often do you have the following:** |  | **Per day** | **Per week** | **Per month** | **Rarely/Never** |
| 500. Alcohol (beer/stout/wine/hard liquor) | 1 serving |  |  |  |  |


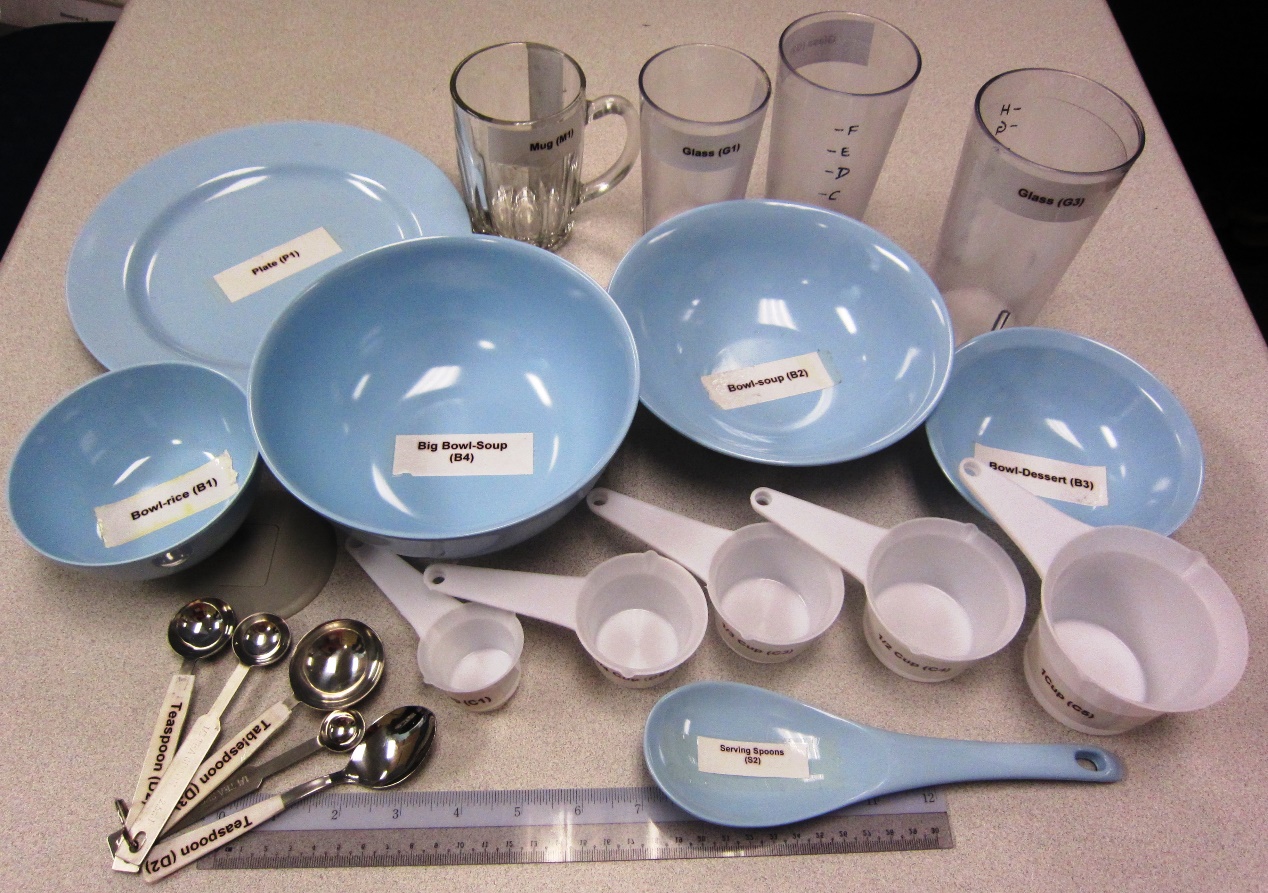

Supplement: Supplementary Text 1 [file aging-12-202277-s002.docx]
