# Supplementary material for: Mental awareness improved mild cognitive impairment and modulated gut microbiome
Source: Aging (Albany NY). 2020 Dec 9;12(23):24371–93. doi: 10.18632/aging.202277 (PMC7762482; doi:10.18632/aging.202277)
Supplement: Supplementary Text 2 [file aging-12-202277-s003.docx]

**Supplementary Text 2: Neuropsychological test battery Forms**

| Subject number: |  |  |  |  |  | SLAS number: |  |  |  |  |  |
| --- | --- | --- | --- | --- | --- | --- | --- | --- | --- | --- | --- |

| Date of assessment: | D | D | - | M | M | - | Y | Y | Y | Y |
| --- | --- | --- | --- | --- | --- | --- | --- | --- | --- | --- |

| Mode of Language: |  |
| --- | --- |

| Name of Assessor: |  |
| --- | --- |

**IMPORTANT:**

Conditions other than cognitive impairment may affect performance on neuropsychological test

Please circle the corresponding number and provide details if the testee has any of the following conditions:

1. Hearing impairment _________________________________________________________________
2. Visual impairment __________________________________________________________________
3. Colour blind ____________________________________________________________________
4. Upper extremity disability _____________________________________________________________
5. Others, please specify _________________________________________________________________

| **General Introduction** |
| --- |

Now we are going to do some different activities and this will take about 45 minutes. Some activities are simple but some are more difficult. Please don’t worry if you find some activities difficult. Most people don’t answer everything correctly or finish everything. Just try your best and if you have any questions, please let me know. Okay?

| **1** | **Rey Auditory Verbal Learning Test (RAVLT)** |
| --- | --- |

| Trial 1:  ***“I am going to read a list of words. Please listen carefully and, when I finish, repeat back as many words as you can. You don’t have to say them in the same order that I do - just repeat back as many words as you can remember, in any order. Okay?***  Read the words at the rate of 1 per second. Record responses in order (write numbers), repetitions (mark with “R”), and intrusions (write word). When participant repeats a word, say: ***“You’ve said that before.”***  Trials 2-5:  ***“I am going to read the list again. When I finish, repeat back as many words as you can, even if you have already said them before. Okay?”***  Trial B:  ***“Now I am going to read a second list of words. This time, repeat back as many words as you can from the second list, in any order. Okay?”***  Recall A:  ***“Do you remember the words from the first list? Now, tell me as many words as you can remember.”***  Scoring: 1 point for each word correctly recalled on each trial. |
| --- |

| **LIST A** | **T1** | **T2** | **T3** | **T4** | **T5** | **LIST B** | **TB** | **REC A** | **LIST A** |
| --- | --- | --- | --- | --- | --- | --- | --- | --- | --- |
| 1 DRUM |  |  |  |  |  | 1 DESK |  |  | 1 DRUM |
| 2 CURTAIN |  |  |  |  |  | 2 RANGER |  |  | 2 CURTAIN |
| 3 BELL |  |  |  |  |  | 3 BIRD |  |  | 3 BELL |
| 4 COFFEE |  |  |  |  |  | 4 SHOE |  |  | 4 COFFEE |
| 5 SCHOOL |  |  |  |  |  | 5 STOVE |  |  | 5 SCHOOL |
| 6 PARENT |  |  |  |  |  | 6 MOUNTAIN |  |  | 6 PARENT |
| 7 MOON |  |  |  |  |  | 7 GLASSES |  |  | 7 MOON |
| 8 GARDEN |  |  |  |  |  | 8 TOWEL |  |  | 8 GARDEN |
| 9 HAT |  |  |  |  |  | 9 CLOUD |  |  | 9 HAT |
| 10 FARMER |  |  |  |  |  | 10 BOAT |  |  | 10 FARMER |
| 11 NOSE |  |  |  |  |  | 11 LAMB |  |  | 11 NOSE |
| 12 TURKEY |  |  |  |  |  | 12 GUN |  |  | 12 TURKEY |
| 13 COLOUR |  |  |  |  |  | 13 PENCIL |  |  | 13 COLOUR |
| 14 HOUSE |  |  |  |  |  | 14 CHURCH |  |  | 14 HOUSE |
| 15 RIVER |  |  |  |  |  | 15 FISH |  |  | 15 RIVER |
| **Correct:** |  |  |  |  |  |  |  |  |  |
| **Repetitions:** |  |  |  |  |  |  |  |  |  |
| **Intrusions:** |  |  |  |  |  |  |  |  |  |

| **2** | **Digit Span** |
| --- | --- |

| **Item** | **Forward: Trial 1** | **Score** | **Forward: Trial 2** | **Score** |  |
| --- | --- | --- | --- | --- | --- |
| **1.** | **1 – 7** |  | **6 – 3** |  |  |
| **2.** | **5 – 8 – 2** |  | **6 – 9 – 4** |  |  |
| **3.** | **6 – 4 – 3 – 9** |  | **7 – 2 – 8 – 6** |  |  |
| **4.** | **4 – 2 – 7 – 3 – 1** |  | **7 – 5 – 8 – 3 – 6** |  |  |
| **5.** | **6 – 1 – 9 – 4 – 7 – 3** |  | **3 – 9 – 2 – 4 – 8 – 7** |  |  |
| **6.** | **5 – 9 – 1 – 7 – 4 – 2 – 8** |  | **4 – 1 – 7 – 9 – 3 – 8 – 6** |  |  |
| **7.** | **5 – 8 – 1 – 9 – 2 – 6 – 4 – 7** |  | **3 – 8 – 2 – 9 – 5 – 1 – 7 – 4** |  |  |
| **8.** | **2 – 7 – 5 – 8 – 6 – 2 – 5 – 8 – 4** |  | **7 – 1 – 3 – 9 – 4 – 2 – 5 – 6 – 8** |  |  |
| **9.** | **8 – 3 – 7 – 4 – 9 – 5 – 2 – 6 – 1 – 5** |  | **3 – 7 – 8 – 1 – 9 – 6 – 5 – 7 – 4 – 2** |  |  |
| **10.** | **6 – 3 – 8 – 5 – 7 – 1 – 9 – 8 – 4 – 9 – 2** |  | **7 – 4 – 5 – 2 – 6 – 9 – 1 – 5 – 7 – 3 – 8** |  |  |
| **11.** | **2 – 7 – 1 – 9 – 8 – 3 – 6 – 4 – 7 – 5 – 2 – 4** |  | **6 – 9 – 2 – 3 – 1 – 7 – 9 – 6 – 8 – 3 – 5 – 4** |  | Longest span: |
|  | **Trial 1 subtotal:** |  | **Trial 2 subtotal:** |  |  |
| **Item** | **Backward: Trial 1** | **Score** | **Backward: Trial 2** | **Score** |  |
| **1.** | **2 – 4** |  | **5 – 7** |  |  |
| **2.** | **6 – 2 – 9** |  | **4 – 1 – 5** |  |  |
| **3.** | **3 – 2 – 7 – 9** |  | **4 – 9 – 6 – 8** |  |  |
| **4.** | **1 – 5 – 2 – 8 – 6** |  | **6 – 1 – 8 – 4 – 3** |  |  |
| **5.** | **5 – 3 – 9 – 4 – 1 – 8** |  | **7 – 2 – 4 – 8 – 5 – 6** |  |  |
| **6.** | **8 – 1 – 2 – 9 – 3 – 6 – 5** |  | **4 – 7 – 3 – 9 – 1 – 2 – 8** |  |  |
| **7.** | **9 – 4 – 3 – 7 – 6 – 2 – 5 – 8** |  | **7 – 2 – 8 – 1 – 9 – 6 – 5 – 3** |  |  |
| **8.** | **6 – 2 – 9 – 7 – 4 – 2 – 8 – 5 – 1** |  | **3 – 8 – 5 – 7 – 1 – 4 – 9 – 6 – 2** |  |  |
| **9.** | **5 – 6 – 3 – 9 – 1 – 7 – 4 – 8 – 5 – 2** |  | **3 – 7 – 4 – 2 – 9 – 2 – 6 – 1 – 5 – 7** |  |  |
| **10.** | **1 – 7 – 4 – 2 – 5 – 1 – 3 – 9 – 8 – 9 – 6** |  | **8 – 2 – 9 – 3 – 7 – 1 – 4 – 2 – 6 – 7 – 5** |  | Longest span: |
|  | **Trial 1 subtotal:** |  | **Trial 2 subtotal:** |  |  |

| Forward Span:  ***“I am going to say some numbers, and when I finish, repeat them after me. Okay?”***  Read the numbers at the rate of 1/s. Record ALL responses. Discontinue after failure of both strings in any set.  Backward Span:  ***“I am going to say some more numbers. But this time when I stop, you must say them in the backwards order. For example, if I say ‘7-1-9’, what would you say?”***  If correct: ***“That’s right.”***  If incorrect: ***“No, you would say ‘9-1-7’. I said ‘7-1-9’, so to say it backwards, you would say ‘9-1-7’. I am going to say more numbers. Remember, you must say them backwards: ‘3-4-8’.”*** If they are incorrect on the second example, correct them and continue with testing.  Scoring: 1 point for each correct response. Record longest span length (most number of digits recalled in any 1 trial). |
| --- |

| **3** | **Colour Trails 1 & 2** |
| --- | --- |

| Pretest: ***“Count aloud from 1 to 25.”*** If participant unable to complete this task, do not administer test.  Colour Trails 1 Practice Trial:  ***“There are different coloured circles with numbers in them. Take this pen and connect the circles by going from 1*** (point to 1)***, to 2*** (point to 2**)*, to 3*** (point to 3)***, and so on, until you reach the end. Connect the circles in the correct order as quickly as you can, without lifting the pen from the paper. If you make a mistake, I will point it out. When I do, move the pen back to the last correct circle and continue from there. The line that you draw must go through the circles. Let’s practice. Put your pen here where this hand tells you to start. When I say ‘begin,’ connect the circles in order as quickly as you can until you reach the circle next to the hand telling you to stop. Ready? Begin. ”***  Begin timing as soon as you detect movement toward the first circle. Monitor participant. Immediately correct errors by instructing participant to return pen to the last correct circle and to proceed from there. Check time on stopwatch whenever participant reaches the next circle - if 10 seconds have elapsed since the last correct response, prompt the participant by pointing to the next correct circle. Stop timing as soon as the pencil first touches the outer part of the last circle. Provide verbal encouragement.  Colour Trails 1 Test Trial:  ***“Now there are more numbers and circles. Connect the circles in order like what you did just now. Work as quickly as you can, and do not lift the pen from the paper as you go. Make sure that your lines go through the circles.*** (Point to the first circle.) ***You will start here and end here*** (point)***. Ready? Begin.”*** After 4 min, ***“Stop.”***  Colour Trails 2 Practice Trial:  ***“There are different coloured circles with numbers in them. This time I want you to connect the circles in order by going from this colour 1*** (point to pink 1**)*, to this colour 2*** (point to yellow 2)***, to this colour 3*** (point to pink 3)***, and so on, until you reach the last number*** (point)***.”***  Point to the example below the box and say:  ***“Notice that the colour changes each time you go to the next number. Work as quickly as you can. Do not lift the pen from the paper once you have started. If you make a mistake, I will point it out. When I do, move the pen back to the last correct circle and continue from there. The line you draw must go through the circles. Do you have any questions? Ok, let’s practice. Put your pen here*** (point)***. When I say ‘begin,’ connect the circles in order as quickly as you can, changing from one colour to the next, until you reach the end*** (point)***. Ready? Begin.”***  Colour Trails 2 Test Trial:  ***“Now there are more numbers and coloured circles. Connect the circles like what you did just now. Work as quickly as you can.*** (Point to the first circle.) ***Start here and end here*** (point)***. Ready? Begin.”*** After 4 min, ***“Stop.”***  Scoring: Record time taken to complete test trials in seconds, number of errors (when participant connects to a circle that is not in the proper numerical sequence or that is not the correct colour), number of near misses (initiation of line towards incorrect circle that is self-corrected before pen connects to incorrect circle), and number of prompts |
| --- |

| **Test Trial** | **Time (s)** | **No. of Errors** | | **No. of Near-Misses** | **No. of Prompts** |
| --- | --- | --- | --- | --- | --- |
| **1** |  |  | |  |  |
|  | | **Colour** | **No.** |  | |
| **2** |  |  |  |  |  |

| **4** | **Block Design** |
| --- | --- |


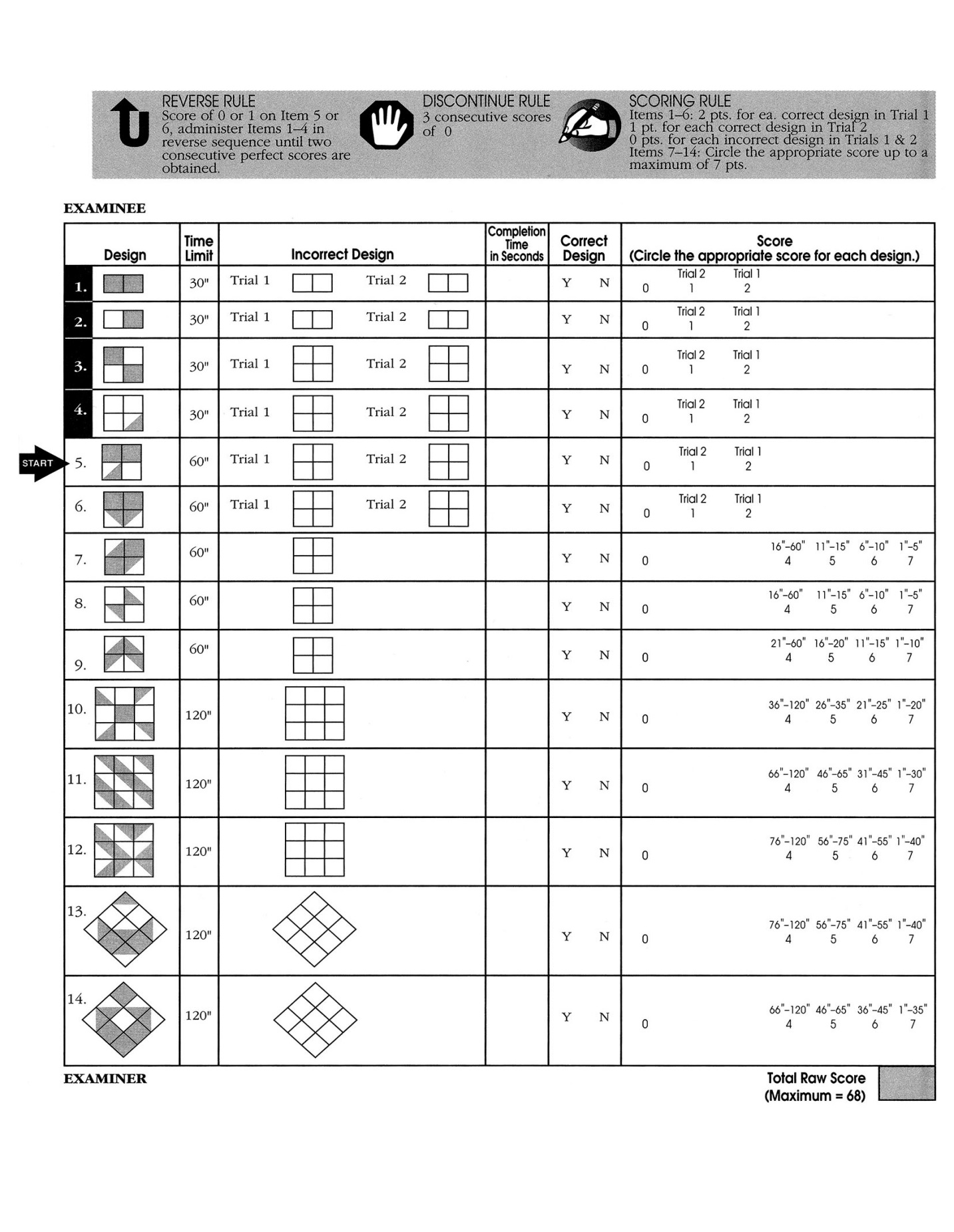


| **5** | **RAVLT – Delayed Recall & Recognition** |
| --- | --- |

| Recall:  ***“Earlier, I read two lists of words to you. I read the first list of words many times. Please tell me as many of those words as you can remember now.”***    Record responses in order (write numbers), repetitions (mark with “R”), and intrusions (write word).  Scoring: 1 point for each word correctly recalled on each trial.  Recognition:  *“****I am going to read you some words. If the word was on the first list, say ‘Yes’ and if it was not, say ‘No’.****”*  Scoring: 1 point for each word correctly identified. Score also false positives. Circle ALL word items that were incorrectly recognized (answer is Y but participant said N, and vice versa). |
| --- |

| **RECALL** | |  | **RECOGNITION** | | | | | |
| --- | --- | --- | --- | --- | --- | --- | --- | --- |
| **LIST A** | **Del A** |  | **Y/N** | **Words** | **Y/N** | **Words** | **Y/N** | **Words** |
| 1 DRUM |  |  |  | **1 BELL** |  | **18 COFFEE** |  | **35 FARMER** |
| 2 CURTAIN |  |  |  | 2 WINDOW |  | 19 MOUSE |  | 36 ROSE |
| 3 BELL |  |  |  | **3 HAT** |  | **20 RIVER** |  | 37 CLOUD |
| 4 COFFEE |  |  |  | 4 BARN |  | 21 TOWEL |  | **38 HOUSE** |
| 5 SCHOOL |  |  |  | 5 RANGER |  | **22 CURTAIN** |  | 39 STRANGER |
| 6 PARENT |  |  |  | **6 NOSE** |  | 23 FLOWER |  | **40 GARDEN** |
| 7 MOON |  |  |  | 7 WEATHER |  | **24 COLOUR** |  | 41 GLASSES |
| 8 GARDEN |  |  |  | **8 SCHOOL** |  | 25 DESK |  | 42 STOCKING |
| 9 HAT |  |  |  | 9 HAND |  | 26 GUN |  | 43 SHOE |
| 10 FARMER |  |  |  | 10 PENCIL |  | 27 CRAYON |  | 44 TEACHER |
| 11 NOSE |  |  |  | 11 HOME |  | 28 CHURCH |  | 45 STOVE |
| 12 TURKEY |  |  |  | 12 FISH |  | **29 TURKEY** |  | 46 NEST |
| 13 COLOUR |  |  |  | **13 MOON** |  | 30 FOUNTAIN |  | 47 CHILDREN |
| 14 HOUSE |  |  |  | 14 TREE |  | 31 BOAT |  | **48 DRUM** |
| 15 RIVER |  |  |  | 15 BALLOON |  | 32 HOT |  | 49 TOFFEE |
|  |  |  |  | 16 BIRD |  | **33 PARENT** |  | 50 LAMB |
| **Correct:** |  |  |  | 17 MOUNTAIN |  | 34 WATER |  |  |
| **Repetitions:** |  |  | **Correct: / 15** | | | | | |
| **Intrusions:** |  |  | **False Positive: / 35** | | | | | |

| **6** | **Semantic Fluency (animals)** |
| --- | --- |

**Now I would like you to name as many animals as you can. Please give me all the animals that you can think of, as fast as you can in one minute. Are you ready? Start.**

|  | **Responses** | **C** | **R** | **I** |  | **Responses** | **C** | **R** | **I** |
| --- | --- | --- | --- | --- | --- | --- | --- | --- | --- |
| 1 |  |  |  |  | 21 |  |  |  |  |
| 2 |  |  |  |  | 22 |  |  |  |  |
| 3 |  |  |  |  | 23 |  |  |  |  |
| 4 |  |  |  |  | 24 |  |  |  |  |
| 5 |  |  |  |  | 25 |  |  |  |  |
| 6 |  |  |  |  | 26 |  |  |  |  |
| 7 |  |  |  |  | 27 |  |  |  |  |
| 8 |  |  |  |  | 28 |  |  |  |  |
| 9 |  |  |  |  | 29 |  |  |  |  |
| 10 |  |  |  |  | 30 |  |  |  |  |
| 11 |  |  |  |  | 31 |  |  |  |  |
| 12 |  |  |  |  | 32 |  |  |  |  |
| 13 |  |  |  |  | 33 |  |  |  |  |
| 14 |  |  |  |  | 34 |  |  |  |  |
| 15 |  |  |  |  | 35 |  |  |  |  |
| 16 |  |  |  |  | 36 |  |  |  |  |
| 17 |  |  |  |  | 37 |  |  |  |  |
| 18 |  |  |  |  | 38 |  |  |  |  |
| 19 |  |  |  |  | 39 |  |  |  |  |
| 20 |  |  |  |  | 40 |  |  |  |  |
|  | | | | | **Total Score (C, R, I):** | |  |  |  |
